# The Impact of Early Diagenesis on Biosignature Preservation in Sulfate Evaporites: Insights From Messinian (Late Miocene) Gypsum

**DOI:** 10.1111/gbi.70007

**Published:** 2024-12-09

**Authors:** Luca Pellegrino, Marcello Natalicchio, Andrea Cotellucci, Andrea Genre, Richard W. Jordan, Giorgio Carnevale, Francesco Dela Pierre

**Affiliations:** ^1^ Dipartimento di Scienze Della Terra Università Degli Studi di Torino Torino Italy; ^2^ Dipartimento di Scienze Della Vita e Biologia Dei Sistemi Università Degli Studi di Torino Torino Italy; ^3^ Faculty of Science Yamagata University Yamagata Japan

**Keywords:** authigenic clays, biosignatures, Mars, Messinian, salt giants, sulfate evaporites

## Abstract

Due to their fast precipitation rate, sulfate evaporites represent excellent repositories of past life on Earth and potentially on other solid planets. Nevertheless, the preservation potential of biogenic remains can be compromised by extremely fast early diagenetic processes. The upper Miocene, gypsum‐bearing sedimentary successions of the Mediterranean region, that formed *ca.* 6 million years ago during the Messinian salinity crisis, represent an excellent case study for investigating these diagenetic processes at the expense of organic matter and associated biominerals. Several gypsum crystals from the Northern Mediterranean were studied by means of destructive and non‐destructive techniques in order to characterize their solid inclusion content and preservation state. In the same crystal, excellently preserved microfossils coexist with strongly altered biogenic remains. Altered remains are associated with authigenic minerals, especially clays. The results demonstrate that a significant fraction of organic matter and associated biominerals (notably biogenic silica) underwent early diagenetic modification. The latter was likely triggered by bottom sulfidic conditions when the growth of gypsum was interrupted. These results have significant implications for the interpretation of the Messinian Salt Giant.

## 1 Introduction

Due to the fast growth rate (tens of μm/day), evaporites represent excellent traps for biogenic material (e.g., Benison 2019; Gibson and Benison 2023; Gill et al. 2023). These chemical sediments may rapidly entomb biogenic particles or biomolecules allowing for their excellent preservation on geological timescales (e.g., Petrichenko, Peryt, and Poberegsky 1997; Fish et al. 2002; Benison 2019; Natalicchio et al. 2022). However, evaporite paleobiology has been so far poorly explored, mostly because of the assumption that evaporites reflect inhospitable conditions for most life forms (e.g., Gibson and Benison 2023). Most studies have focused on halite (NaCl) (e.g., Schubert, Lowenstein, and Timofeeff 2009; Lowenstein, Schubert, and Timofeeff 2011; Gibson and Bodman 2021; Gibson 2022). In contrast, sulfate evaporites were less explored, although these salts and in particular gypsum (CaSO_4_·2H_2_O) and mirabilite (Na_2_SO_4_·10H_2_O) are also known to preserve organic material (e.g., Benison and Karmanocky III 2014; Schopf et al. 2012; Dela Pierre et al. 2015; Pellegrino et al. 2021; Natalicchio et al. 2022; Gill et al. 2023). Since sulfate‐rich sedimentary rocks were observed on the Martian surface and are one of the sampling targets of the Rover Perserverance in Jezero crater, the characterization of biosignatures and their preservation potential in terrestrial analogs (especially gypsum) may have relevant astrobiological implications (e.g., Langevin et al. 2005; Vaniman et al. 2018; Benison et al. 2024; Siljeström et al. 2024; Stack et al. 2024). An important aspect that needs to be considered is the possible impact of diagenetic processes on biosignature preservation. While the influence of burial diagenesis has already been discussed (e.g., Shkolyar and Farmer 2018), little is known about very early diagenetic processes occurring immediately after deposition of biogenic material but before its definitive entrapment in the crystal.

Basin‐wide evaporite deposition took place in the Mediterranean basin during the so‐called Messinian salinity crisis (MSC: 5.97–5.33 Ma), following the partial isolation of the Mediterranean Sea from the Atlantic Ocean (e.g., Hsü, Ryan, and Cita 1973; Krijgsman et al. 1999). The sedimentary products of the MSC consist of more than 1 million km^3^ of evaporites (carbonate minerals, gypsum, anhydrite, and halite) that were deposited in both marginal and deep basinal areas, the latter buried under the abyssal plains of the modern Mediterranean Sea (e.g., Roveri et al. 2014). Messinian gypsum is represented by three stratigraphic units (e.g., CIESM 2008): (i) the Primary Lower Gypsum (PLG) unit, the object of this study, deposited during the first phase of the MSC (5.97–5.60 Ma) in marginal basins and consisting of bottom‐grown primary gypsum beds interbedded with organic‐rich shales; (ii) the Resedimented Lower Gypsum unit deposited during the second phase of the MSC (5.60–5.55 Ma) and consisting of clastic gypsum and halite; (iii) the Upper Gypsum unit, composed of bottom‐grown gypsum deposited during the third phase of the MSC (5.55–5.33 Ma).

**FIGURE 1:**
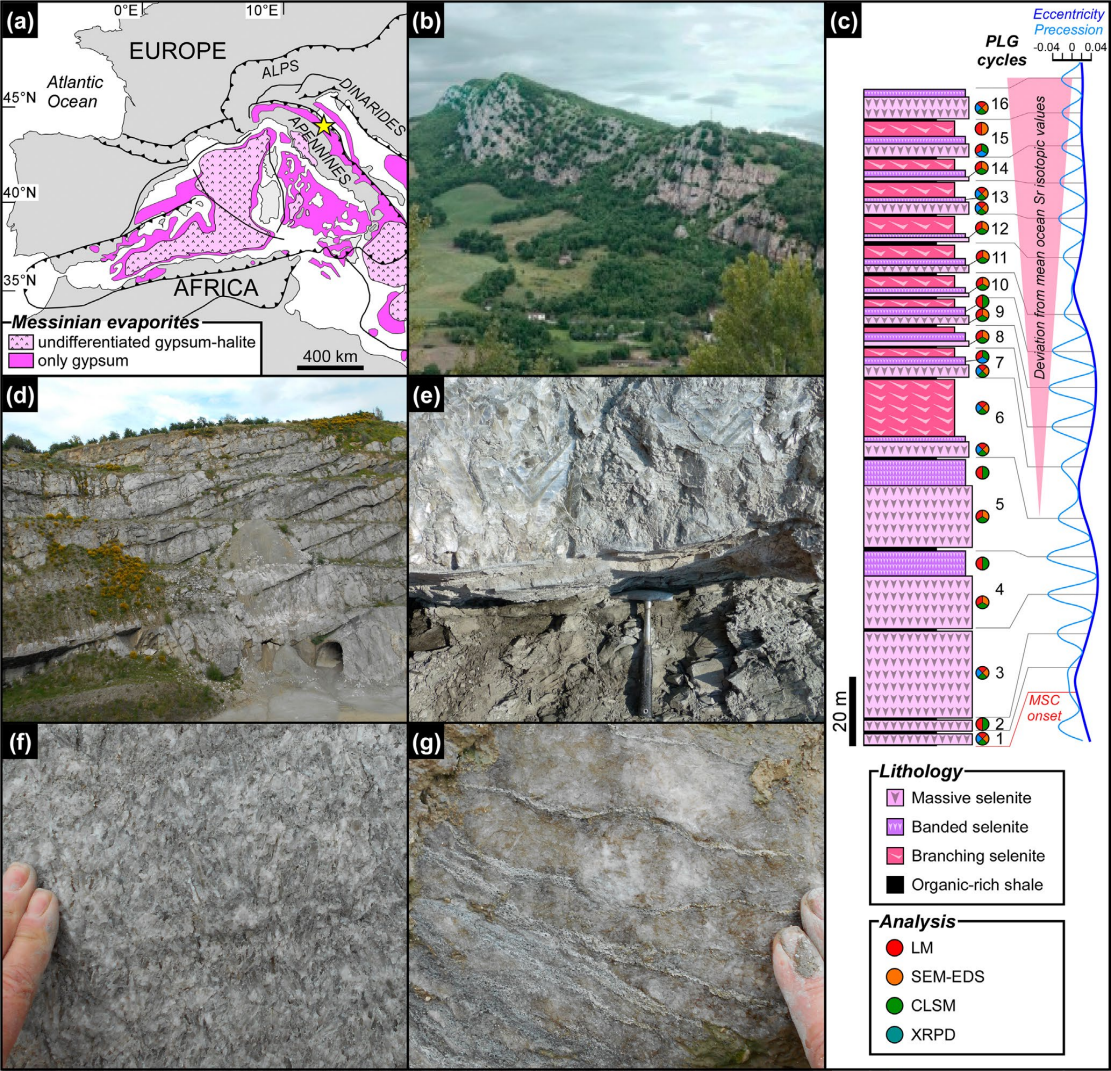
Geological and stratigraphic setting of the Vena del Gesso basin. (a) Distribution of Messinian evaporitic deposits in the Western Mediterranean region (modified from Lugli et al. 2010); the yellow star indicates the study area. (b) Panoramic view of the Vena del Gesso ridge. Note the cyclic stacking pattern consisting of the alternation of thin organic‐rich shales and thick gypsum beds. (c) The Vena del Gesso section (modified from Guibourdenche et al. 2022) and tuning with the astronomical curve (Laskar et al. 2004). Samples and applied analytical techniques are indicated; CLSM = confocal laser scanning microscopy, LM = light microscopy, SEM‐EDS = scanning electron microscopy coupled to X‐ray energy dispersive spectroscopy, XRPD = X‐ray powder diffraction. (d) Panoramic view of the Monte Tondo quarry. (e) Contact between organic‐rich shale and massive selenite lithofacies. (f) Banded selenite lithofacies. (g) Branching selenite lithofacies.

The widespread occurrence of microfossils and organic compounds in Messinian gypsum confirmed its high biosignature preservation potential (e.g., Panieri et al. 2008, 2010; Schopf et al. 2012; Dela Pierre et al. 2015; Carnevale et al. 2019; Costanzo et al. 2019; Pellegrino et al. 2021; Natalicchio et al. 2022). However, the effects of early diagenetic processes on such potential still need to be evaluated. A recent study focused on non‐evaporitic sediments (dolomite‐rich mudstones and diatomaceous shales) of the PLG unit from Northern Italy showed that biogenic silica and organic matter were rapidly converted into authigenic clay minerals and dolomite, leaving in the sedimentary record only ghosts of the original communities of biosiliceous primary producers (diatoms) and their lipid biomarkers (e.g., Pellegrino et al. 2023). Such findings have suggested that the MSC sedimentary record is actually affected by a diagenetic bias that compromised the preservation of a significant fraction of the biotic assemblage (e.g., Mancini et al. 2022; Pellegrino et al. 2023). Considering that biogenic silica and associated organic matter were observed in Messinian gypsum (e.g., Pellegrino et al. 2021), did these processes also impact biosignature preservation in this lithology? In this paper we provide a multidisciplinary study of the solid inclusions preserved in gypsum crystals of the PLG unit from the Northern Mediterranean region, focusing on the Vena del Gesso section (Figure 1a,b). The main aim is to elucidate the early diagenetic processes that occurred at the expense of organic matter and associated biominerals, notably diatomaceous biogenic silica, before their definitive entrapment in gypsum.

## 2 The Primary Lower Gypsum Unit

### 2.1 Stratigraphy and Sedimentology of the PLG Unit

**FIGURE 2:**
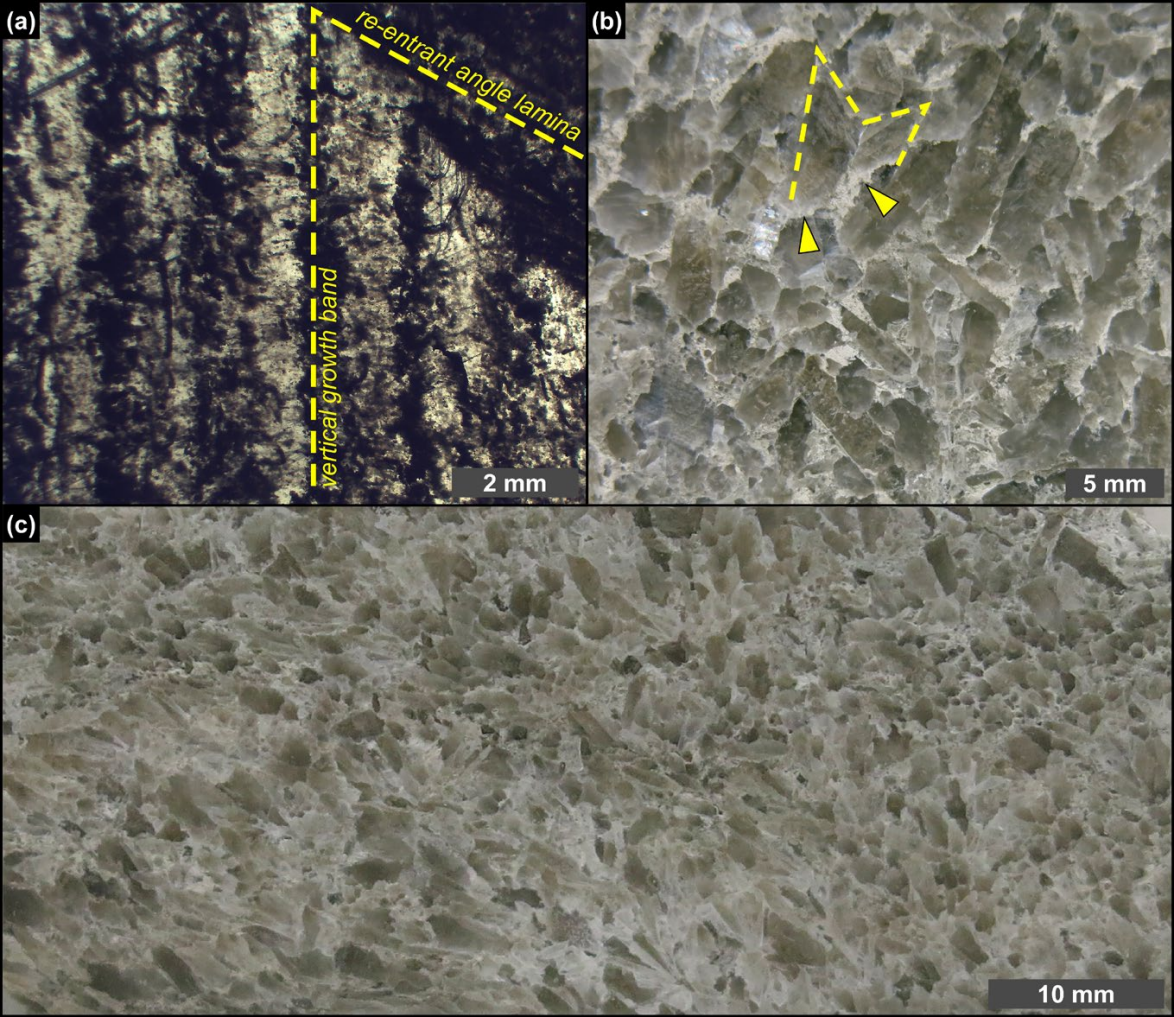
Petrographic overview. (a) Polished thin section of a massive selenite crystal; note that the laminae in the re‐entrant angle can be traced in the vertical growth bands (1st PLG cycle). (b) Polished slab of banded selenite showing interlocked cm‐sized crystals; dotted line highlights a twinned selenite crystal surrounded by a whitish carbonate matrix (arrowheads) (4th PLG cycle). (c) Polished slab of branching selenite showing mm‐sized crystals interspersed in a carbonate‐rich matrix (6th PLG cycle).

The PLG unit consists of up to 16 precession‐controlled lithological cycles formed by organic‐rich shale/gypsum couplets (e.g., Lugli et al. 2010). Tuning of the lithological cycles to the astronomical curve allows the precise dating of each cycle and bed‐by‐bed correlation of the PLG unit at the Mediterranean scale (Figure 1c) (e.g., Krijgsman et al. 1999; Laskar et al. 2004; Roveri et al. 2014). Organic‐rich shales form dm‐ to m‐thick layers that were deposited under a more humid climate at precession minima/insolation maxima (e.g., Lugli et al. 2010; Figure 1c,d). Gypsum beds range in thickness from 4 to 30 m and formed under a more arid climate at precession maxima/insolation minima (e.g., Lugli et al. 2010; Figure 1c,d). Their lateral and vertical stacking pattern reflects the progressive shallowing of the basin (e.g., Lugli et al. 2010; Natalicchio et al. 2021). Different lithofacies were distinguished (Figures 1e–g and 2), according to the shape, size, and orientation of the gypsum crystals, in turn reflecting variable conditions of growth (e.g., Lugli et al. 2010). The gypsum beds of the first three cycles consist exclusively of the massive selenite lithofacies (Figure 1e), represented by large (m‐ to dm‐sized), bottom‐grown and vertically oriented twinned selenite crystals (arrow head or swallow tail twins) following the 100 and $\bar{1}$01 contact twinning laws (e.g., Cotellucci et al. 2023). Their large size suggests a low degree of supersaturation of the brines that allowed the crystals to grow larger (e.g., Lugli et al. 2010; Ortí 2011). The twins display an internal lamination in the reentrant angle, marked by the alternation of mm‐ to submm‐thick, turbid (solid inclusion‐rich) and clear (solid inclusion‐poor) laminae (Figure 2a). Such lamination possibly reflects short‐term (seasonal) climate oscillations causing cyclic changes of gypsum saturation state in bottom waters (e.g., Dela Pierre et al. 2015; Reghizzi et al. 2018). During the more humid season, gypsum growth was interrupted by enhanced freshwater input into the basin, resulting in the accumulation of sediment and biogenic material on the top of the crystals. This material was subsequently entrapped by the rapid growth of a mm‐thick gypsum lamina during the more arid season (e.g., Natalicchio et al. 2021).

In the Vena del Gesso basin, the massive selenite lithofacies is followed upward by the banded selenite lithofacies, starting from the gypsum bed of the fourth cycle (Figure 1c,f) (e.g., Vai and Ricci Lucchi 1977; Lugli et al. 2010). The banded selenite consists of palisades of cm‐ to dm‐sized twinned crystals separated by mm‐thick carbonate layers indicating periodical interruptions of gypsum growth (Figure 2b) (e.g., Lugli et al. 2010). The smaller‐sized crystals point to a higher degree of brine supersaturation during peaks of aridity, highlighting crystal nucleation and consequent competition (e.g., Lugli et al. 2010). Gypsum crystals show a distinctive dark core enriched in solid inclusions but not a clearly defined lamination as in the massive selenite lithofacies. From the sixth gypsum bed upwards, massive and banded selenite are followed by the branching selenite lithofacies (Figure 1c,g). This lithofacies is characterized by inclined or horizontally oriented mm‐sized selenite crystals occasionally enriched in solid inclusions (Figure 2c). The crystals are embedded in a dolomite‐rich matrix and organized in nodular aggregates (e.g., Lugli et al. 2010; Natalicchio et al. 2021). The appearance of this lithofacies coincides with a detachment of the Sr isotope ratios (^87^Sr/^86^Sr) from global ocean values (Figure 1c), reflecting the gradual restriction of the basin with consequent reduced inflows of Atlantic water and increased sensitivity to continental runoff (e.g., Reghizzi et al. 2018).

### 2.2 Paleobiology of the PLG Unit

Previous studies have showed that the pelitic sediments of the PLG unit contain fossilized fish remains, scattered calcareous nanno‐ and microfossils, diatoms, dinoflagellates, and plants, representing a marine to brackish environment and a subtropical humid terrestrial ecosystem (e.g., Bertini and Martinetto 2008; Dela Pierre et al. 2014; Lozar et al. 2018; Carnevale et al. 2019; Carnevale and Schwarzhans 2022; Mancini et al. 2022; Pellegrino et al. 2023). Organic geochemical data suggest a stratified water column influenced by the input of terrestrial organic matter and densely populated by a phototrophic community composed of diatoms, dinoflagellates, and cyanobacteria—these latter likely associated with diatoms (diatom‐diazotrophic associations), methanogens, and ciliates (e.g., Sinninghe Damsté et al. 1995; Isaji et al. 2019). The pigment inventory consists of carotenoids and degradation products of chlorophylls and bacteriochlorophylls, like chlorins and porphyrins (e.g., Sinninghe Damsté et al. 1995; Mawson and Keely 2008). In particular, the occurrence of isorenieratane, a carotenoid possibly sourced from green sulfur bacteria, suggests the periodic establishment of photic zone euxinia during shale deposition (e.g., Keely et al. 1995; Kenig et al. 1995; Schaeffer et al. 1995; Sinninghe Damsté et al. 1995).

Studies on the paleobiological content of the gypsum beds were focused on the massive selenite lithofacies, because of the larger size of the crystals enabling more detailed observations (e.g., Vai and Ricci Lucchi 1977; Panieri et al. 2008, 2010; Schopf et al. 2012; Dela Pierre et al. 2015; Pellegrino et al. 2021; Natalicchio et al. 2022). The most abundant components are filamentous microfossils, > 40–100 μm across and up to 2 mm long, first attributed to remains of brine shrimp fecal pellets, algae, or cyanobacteria (e.g., Schreiber and Decima 1976; Vai and Ricci Lucchi 1977; Rouchy and Monty 2000; Panieri et al. 2008, 2010). The assignment of the filaments to mat‐forming benthic phototrophs would constrain the depth of gypsum growth to the photic zone (shallower than 200 m; e.g., Lugli et al. 2010). More recent investigations, including the study of lipid biomarkers suggested that the filaments represent fossils of sulfide‐oxidizing bacteria (Schopf et al. 2012; Dela Pierre et al. 2015; Natalicchio et al. 2022). Such an assignment does not provide any depth constraint, since these prokaryotes can live in a wide range of water depth, from bathyal to peritidal settings (e.g., Bailey et al. 2009). On the other hand, it suggests that gypsum formed in a stratified basin temporarily typified by bottom water oxygen depletion. Under these circumstances, intense organoclastic sulfate reduction produced high fluxes of hydrogen sulfide necessary to sustain sulfide‐oxidizing bacteria at the sea floor (e.g., Natalicchio et al. 2022). Evidence of this interplay is witnessed by the occasional occurrence of filaments entirely coated with hollow dolomite microcrystals, whose formation was induced by sulfate‐reducing bacteria (e.g., Vasconcelos et al. 1995; Bontognali et al. 2010; Panieri et al. 2010; Dela Pierre et al. 2015). The oxidation of reduced sulfur species by sulfide‐oxidizing bacteria may have even played a role in gypsum precipitation, promoting a transient increase of the concentration of sulfate ions within the benthic microbial mats (e.g., Aloisi et al. 2022; Guibourdenche et al. 2022). Other common components of the massive selenite lithofacies are diatom remains. In the lower two gypsum beds of the PLG unit, these microfossils are mostly represented by nano‐sized planktonic forms indicative of brackish to marine conditions in the upper water column, with subordinated benthic‐epiphytic taxa probably transported by river runoff or currents (e.g., Pellegrino et al. 2021). These diatoms were probably a source of organic matter exploited by heterotrophic benthic bacteria involved in the biogeochemical sulfur cycle (e.g., Pellegrino et al. 2021).

In contrast, no detailed studies deal with the paleobiological content of the banded and branching selenite lithofacies. However, high contents of microbial dolomite, consisting of hollow microcrystals with negative δ^13^C values, occur in the branching selenite lithofacies pointing to a great availability of organic matter fueling bacterial sulfate reduction in temporarily anoxic pore waters (e.g., Natalicchio et al. 2021).

## 3 Materials and Methods

### 3.1 Studied Section

The studied section is located in the Vena del Gesso basin in the Northern Apennines (Figure 1a). The outcropping sedimentary succession (Figure 1b) forms a NW‐SE striking continuous belt elongated for approximately 15 km (e.g., Vai and Ricci Lucchi 1977; Roveri et al. 2003). The sedimentary succession starts with siliciclastic turbidites (Marnoso Arenacea Fm., Langhian‐Messinian) passing upwards to organic‐rich shales (informally named Ghioli di Letto, lower Messinian) (e.g., Roveri et al. 2003). This unit is overlain by the PLG unit, that consists of 16 lithological cycles made up of shale/gypsum couplets (Figure 1c,d) (e.g., Vai and Ricci Lucchi 1977; Roveri et al. 2003). The evaporitic succession is unconformably overlain by brackish water siliciclastic sediments (Colombacci Fm., late Messinian) deposited between 5.55 and 5.33 Ma, in turn topped by deep‐water marine mudstones (Argille Azzurre Fm., Lower Pliocene) which record the end of the Messinian salinity crisis (Roveri et al. 2003).

Thirty gypsum samples were collected in 2019 from the PLG unit exposed in the Monticino (44°13′29″ N, 11°45′43″ E) and Monte Tondo (44°15′04″ N; 11°40′13″ E) quarries (see Guibourdenche et al. 2022). The samples were observed by petrographic and confocal laser scanning microscopes (CLSM), as well as a scanning electron microscope (SEM). Electron dispersive spectroscopy (EDS) and bulk X‐ray powder diffraction (XRPD) were performed for elemental and mineralogical characterization, respectively (Figure 1c). Raman spectroscopy was also applied on one sample.

### 3.2 Petrographic Observation and Quantification of Solid Inclusions

Petrographic observations were conducted on 22 samples of the massive selenite lithofacies, on five samples of the banded selenite lithofacies and two samples of the branching selenite lithofacies (Figure 1c). For the massive selenite lithofacies, twinned crystals were split along the cleavage plane using a razor blade. The resulting subsamples were gently polished with grinding papers. The procedure was slightly modified for the banded and branching selenite lithofacies, due to the smaller size of the crystals. The samples were firstly broken with a pestle in order to obtain fragments of a few cm^3^. The resulting subsamples were firstly consolidated with epoxy resin, then cut with a circular saw and finally thinned and polished. All the subsamples from the three lithofacies were placed onto a glass slide and observed in transmitted light with an optical microscope equipped with a digital camera (Leica Microsystems, Wetzlar, Germany) at the Department of Earth Sciences, University of Torino. In addition, selected samples of banded and branching selenite were cut with a circular saw and ground in order to obtain polished slabs.

The solid inclusion content has only been semi‐quantitatively estimated except for diatom remains, which represent the most easily distinguishable fraction of gypsum solid inclusions. The relative abundance of diatom remains preserved in gypsum was estimated with the optical microscope: 30 randomly selected fields of view of 19 thinned and polished subsamples were observed at 500×, counting all visible diatom remains.

### 3.3 Confocal Laser Scanning and Electron Microscope Investigation

The solid inclusions of gypsum samples were further characterized by confocal microscopy (TCS‐SP2; Leica Microsystems, Wetzlar, Germany) and scanning electron microscopy coupled to energy dispersive X‐ray spectroscopy (SEM‐EDS; JSM IT300LV, JEOL Limited, Tokyo, Japan; Tescan Vega, Tescan, Brno, Czech Republic) at the Department of Life Sciences and Systems Biology and at the Department of Earth Sciences of the University of Torino, respectively. For confocal imaging, additional subsamples of the three lithofacies (Figure 1c) were freshly broken and observed using either a 20× or a 63× water immersion objective; autofluorescence spectral analyses were performed on selected solid inclusions, by exciting the samples with the 488 nm line of an argon laser and recording the emitted fluorescence between 510 and 710 nm. For SEM‐EDS analysis, freshly broken subsamples (Figure 1c) were dissolved onto a nitrocellulose filter following the method described by Pellegrino et al. (2021). A portion of each filter was then cut and glued onto an aluminum stub, carbon‐ or gold‐coated, and analyzed with an SEM‐EDS.

### 3.4 Raman Spectroscopy and Bulk Mineralogical Analysis

Micro‐Raman spectra were acquired with an integrated micro/macro‐Raman LABRAM HRVIS (Horiba Jobin Yvon Instruments) of the Interdepartmental Center “G. Scansetti” at the Department of Earth Sciences of the University of Torino. Excitation line at 532 nm (solid‐state Nd laser and 80 mW of emission power) was used with Edge filters and a grating of 600 grooves/mm. Calibration was performed using the 520.6 cm^−1^ Si band.

X‐ray powder diffraction analyses were conducted on 10 samples (7 from the massive selenite, 2 from the banded selenite, and 1 from the branching selenite; Figure 1c). In order to concentrate the clay fraction, a dissolution procedure for removing gypsum was setup (see Data S1 for details). The identification and quantification of clay minerals was performed by a Rigaku Miniflex 600 XRD system (CuKα radiation) operating at 40 kV and 15 mA at the Department of Earth Sciences of the University of Torino.

## 4 Results

### 4.1 Types of Solid Inclusions

**FIGURE 3:**
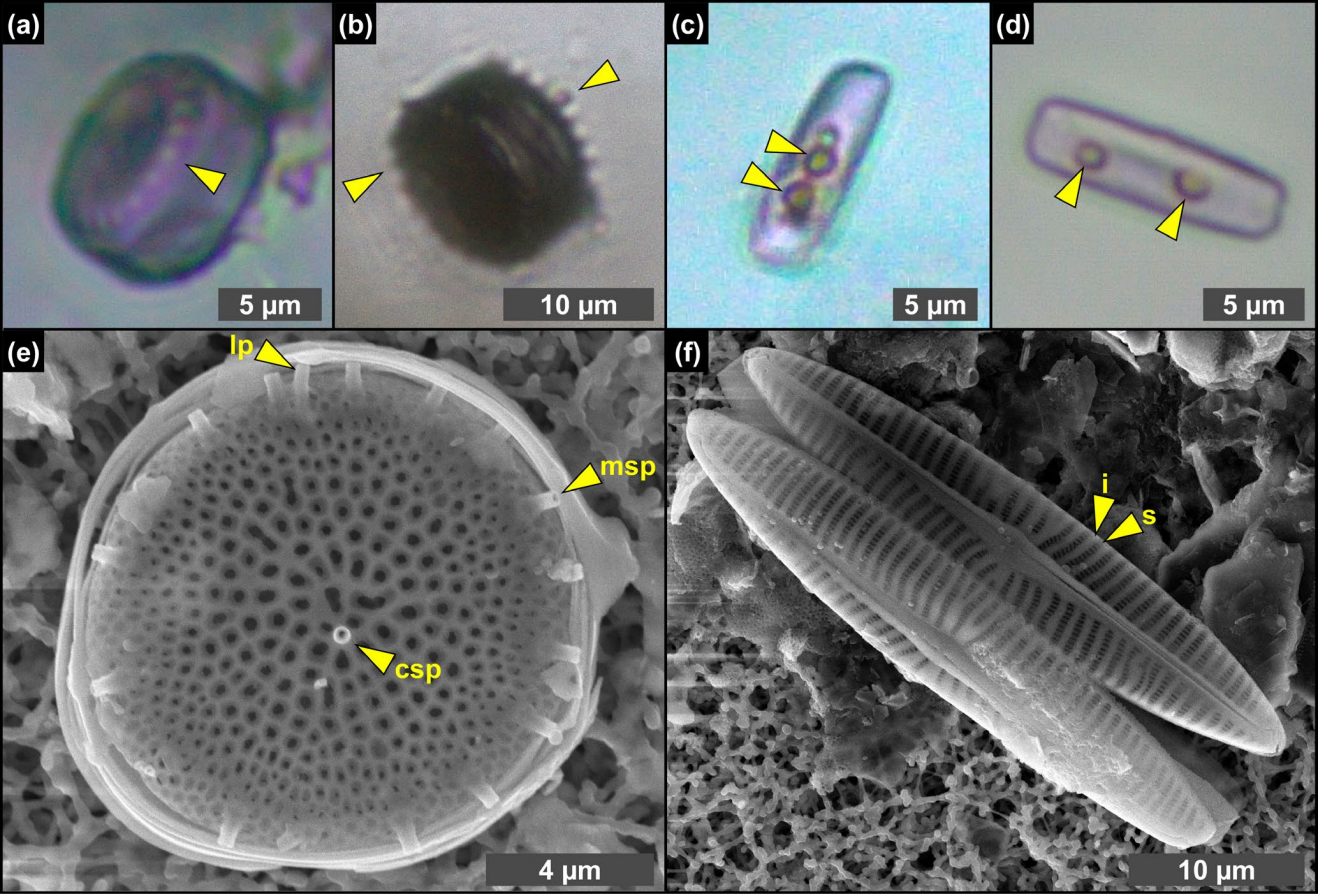
Transmitted light (a–d) and SEM (e, f) photomicrographs of excellently preserved diatom remains from the massive selenite lithofacies. (a, b) Whole frustules of thalassiosiroid diatoms with discernible valve processes, indicated by arrowheads (1st PLG cycle). (c, d) Whole frustules of naviculoid diatoms bearing roundish globules, indicated by arrowheads (5th and 14th PLG cycle). (e) Valve of *Thalassiosira* sp., showing the preservation of central and marginal strutted processes (csp and msp, respectively) and labiate process (lp). (f) Naviculoid diatom remains showing the perfect preservation of valve ornamentation, consisting in the alternation of striae (s) and interstriae (i) (1st PLG cycle).

**FIGURE 4:**
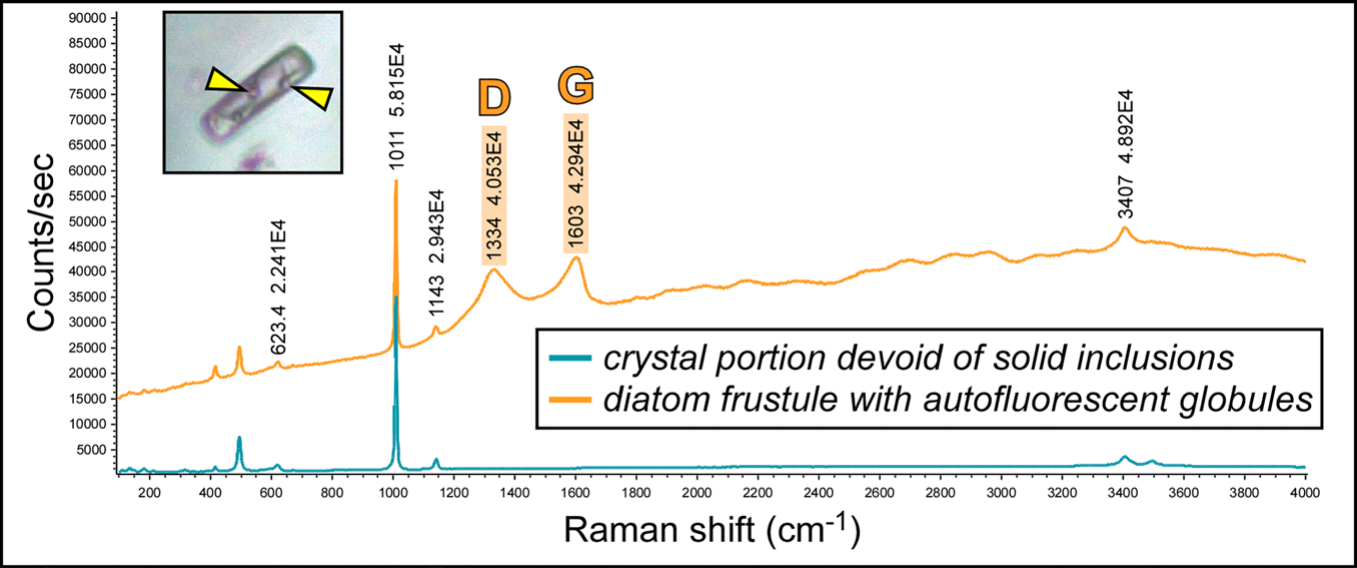
Raman spectra of a limpid portion of twinned crystal and of a diatom frustule including autofluorescent globules, indicated by arrowheads (5th PLG cycle); note two prominent peaks at about 1300 and 1600 cm^−1^ (D‐ and G‐band, respectively). The peak at about 1010 cm^−1^ indicates gypsum.

**FIGURE 5:**
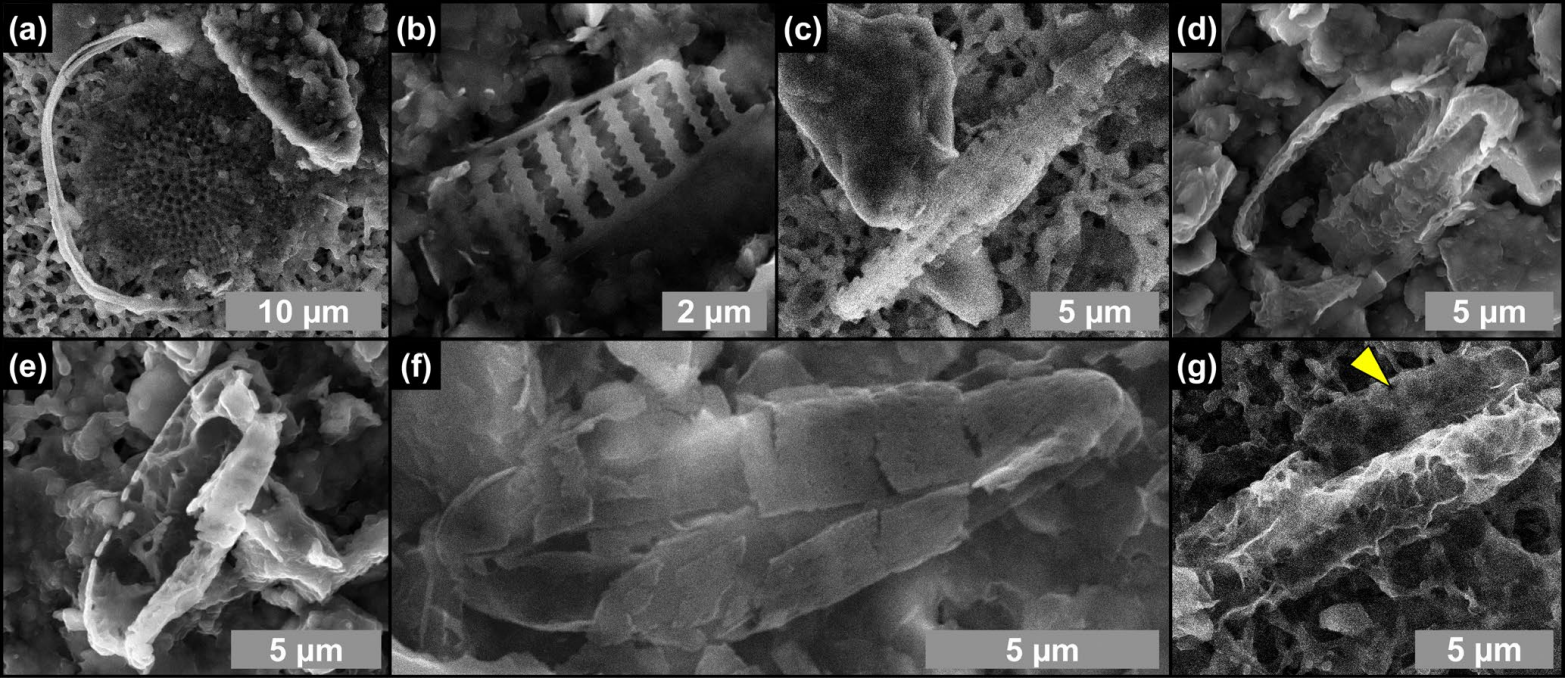
SEM photomicrographs of altered diatom remains from the massive selenite lithofacies. (a–f) Partially dissolved and clay‐coated valves (1st, 4th, 5th and 12th PLG cycle). (g) Naviculoid valve completely replaced by authigenic clays; the other valve, indicated by arrowhead, is still recognizable (5th PLG cycle).

**FIGURE 6:**
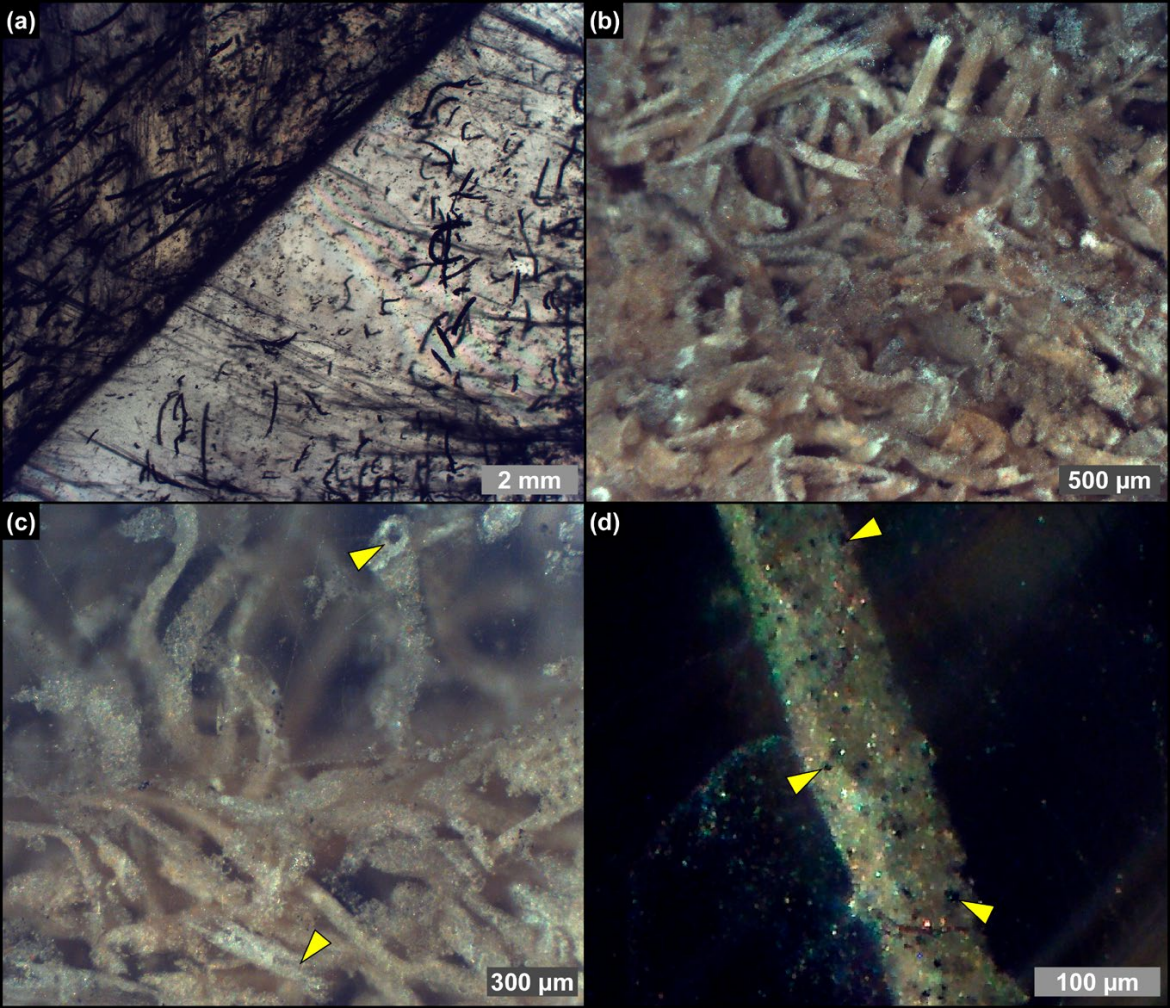
Transmitted (a) and reflected (b–d) light photomicrographs of filamentous microfossils in twinned crystals of the massive selenite lithofacies. (a) Filamentous microfossils distributed along the re‐entrant angle and the vertical growth bands (4th PLG cycle). (b) Dense accumulation of filaments (2nd PLG cycle). (c) Detail of the filaments; note that filaments are hollow (arrowheads) (2nd PLG cycle). (d) Detail of a filament; the opaque grains (arrowheads) correspond to iron sulfides (2nd PLG cycle).

**FIGURE 7:**
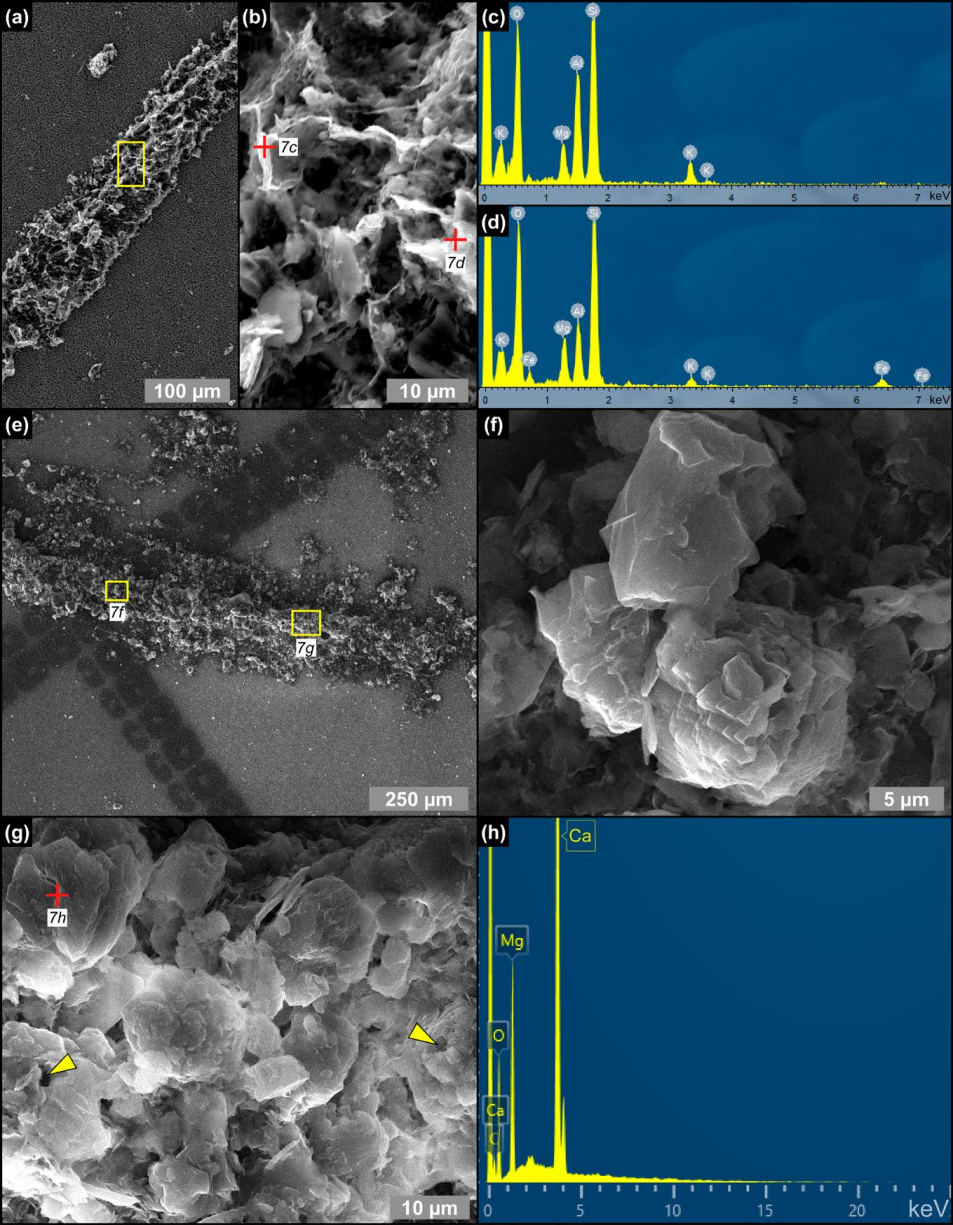
SEM photomicrographs and EDS spectra of filamentous microfossils from the massive selenite lithofacies. (a) Clay‐coated filament, (b) detail, and (c, d) corresponding EDS spectra (5th PLG cycle). (e) Clay‐ and dolomite‐coated filament, (f, g) details, and (h) corresponding EDS spectrum; in (g) arrowheads point to hollow microcrystals (9th PLG cycle).

**FIGURE 8:**
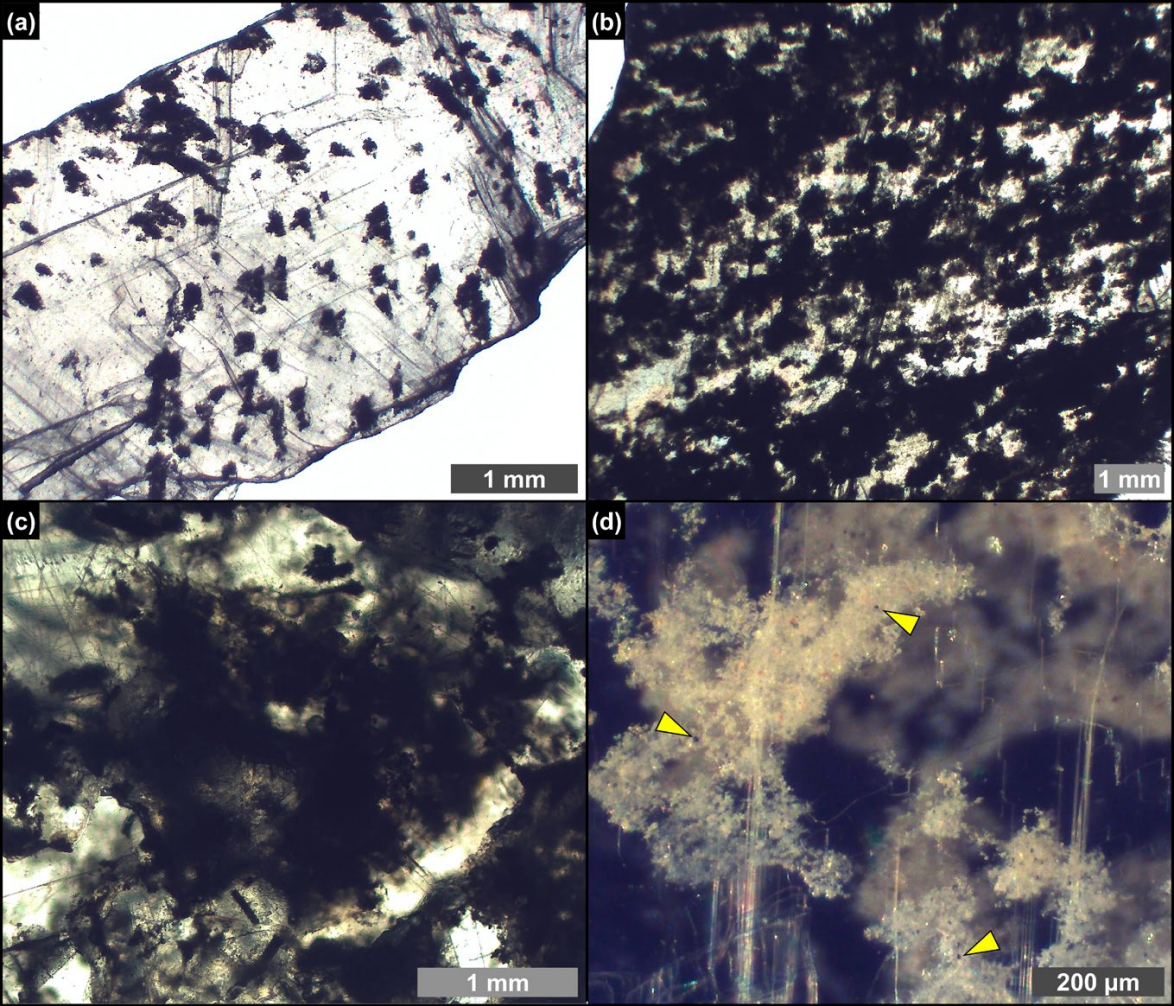
Transmitted (a–c) and reflected light (d) photomicrographs of floccules entrapped in crystals from the massive and branching selenite lithofacies. (a) Widely spaced floccules (massive selenite—7th PLG cycle). (b) Closely spaced floccules (massive selenite—6th PLG cycle). (c) Detail on a floccule from branching (branching selenite—6th PLG cycle). (d) Pale brownish color of floccules in reflected light; the opaque grains (arrowheads) correspond to iron sulfides (massive selenite—9th PLG cycle).

**FIGURE 9:**
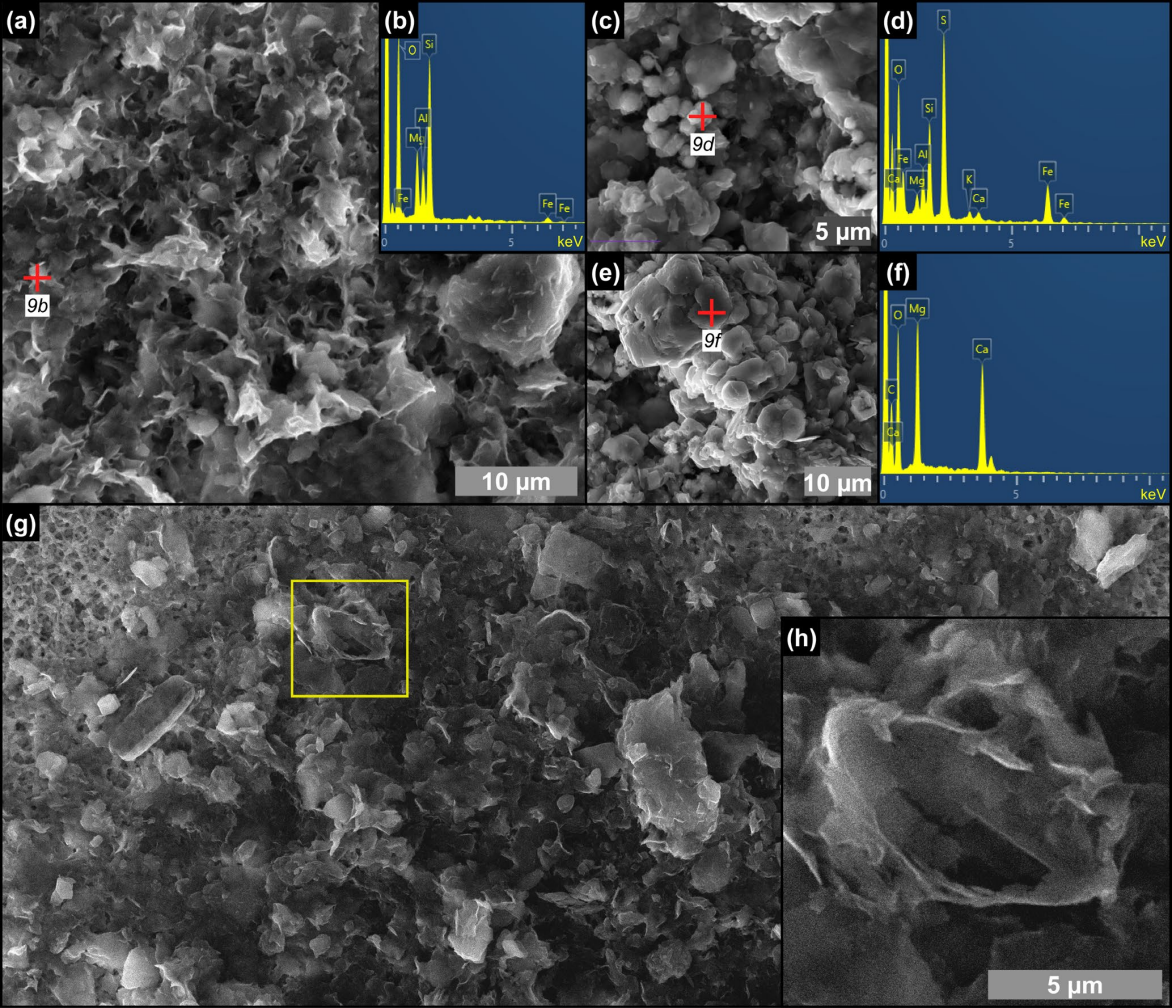
SEM photomicrographs and EDS spectra of the content of floccules from the massive selenite lithofacies. (a) Flaky clays and (b) corresponding EDS spectrum (9th PLG cycle). (c) Pyrite grains associated with clays and (d) corresponding EDS spectrum. (e) Dolomite microcrystals and (f) corresponding EDS spectrum (6th PLG cycle). (g, h) Altered remains of naviculoid diatoms (4th PLG cycle).

**FIGURE 10:**
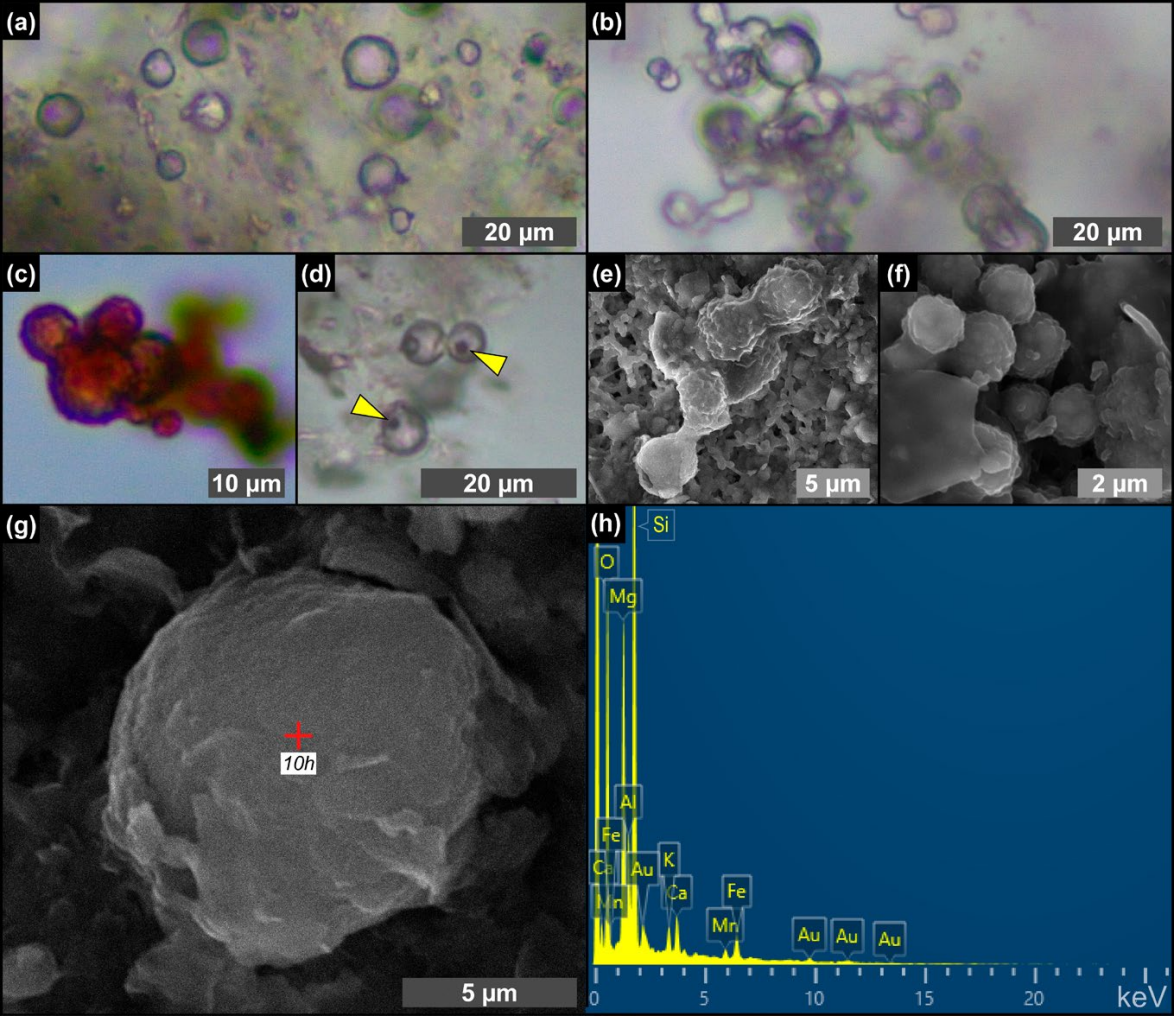
Transmitted light (a–d) and SEM (e–g) photomicrographs and EDS spectrum (h) of spheroids from the massive selenite lithofacies. (a) Isolated spheroids (12th PLG cycle). (b) Chain‐forming spheroids (8th PLG cycle). (c) Reddish spheroids (4th PLG cycle). (d) Reddish button‐like particles, indicated by arrowheads (5th PLG cycle). (e, f) Cluster of very small spheroids inside a floccule (14th PLG cycle). (g) Isolated spheroid in a floccule and (h) corresponding EDS spectrum (12th PLG cycle); Au peaks are related to sample coating.

Three main types of solid inclusions have been distinguished in the studied crystals: (i) microfossils (Figures 3, 4, 5, 6, 7), that is, fossilized remains with a distinct morphology still attributable to a precursor organism; (ii) organic‐ and clay‐rich floccules (Figures 8 and 9); (iii) spheroidal objects of unclear origin (Figure 10).

#### 4.1.1 Microfossils

Microfossils were mostly observed in the twinned selenite crystals of the massive selenite lithofacies, whereas in the banded and branching selenite lithofacies they are rare. They are mostly represented by diatom remains (Figures 3, 4, 5) and filaments (Figures 6 and 7). Locally, other unidentified microalgae (e.g., putative dinoflagellates and green algae), ciliates, pollen grains, and trichomes were found in gypsum crystals from all the analyzed lithofacies.

Diatom remains are more abundant in the lower part of the section, especially in the first and fifth gypsum beds. The assemblage comprises both centric and pennate raphid taxa. Centric diatom remains dominate the assemblage in the first gypsum bed, are less abundant in the second one and are no longer recorded from the third one upward. They are represented by marine to brackish planktonic taxa, mostly thalassiosiroids (both nano‐sized, i.e., < 20 μm, and larger specimens; Figure 3a,b), some remains of chain‐forming *Chaetoceros* vegetative cells, and scattered remains of rhizosolenioids. Pennate raphid diatoms consist of benthic taxa, mostly represented by nano‐sized naviculoids (Figure 3c,d); their abundance spikes in the lower part of the fifth gypsum bed (massive selenite lithofacies). A subordinate pennate diatom component is represented by nitzschioids, *Surirella* spp., *Entomoneis* spp. and scattered amphoroids; large specimens of *Lampriscus* sp. and chains of *Rhabdonema* cf. *adriaticum* have been identified only in the first gypsum bed.

Along the stratigraphic section the state of preservation of diatom remains is variable, and excellently preserved microfossils coexist in the same samples with strongly altered remains. The best‐preserved remains consist of single valves or even whole frustules of both centric and pennate diatoms (Figure 3). These remains do not show evidence of opal dissolution (Figure 3e,f). Very delicate structures, such as thalassiosiroid labiate and strutted processes (Figure 3a,b,e) and naviculoid striae and interstriae (Figure 3f) are still intact. Some of these specimens, especially in the naviculoid group, are typified by tiny globules (Figure 3c,d), a few μm across, that appear reddish to yellowish in transmitted light. The carbonaceous composition of the globules was revealed by Raman spectroscopy (Figure 4), which highlighted two broad bands centered at 1334 and 1603 cm^−1^, corresponding to the disordered (D) band and the ordered (G) band of amorphous carbonaceous material, respectively (e.g., Frezzotti, Tecce, and Casagli 2012; Dela Pierre et al. 2014). In great contrast, altered diatom remains consist of heavily corroded valves coated with Mg‐, K‐ and Fe‐rich aluminosilicates (Figure 5a–f) which in some cases completely replaced the original opaline tests (Figure 5g).

Filaments, recently interpreted as remains of sulfide‐oxidizing bacteria (e.g., Schopf et al. 2012; Dela Pierre et al. 2015; Natalicchio et al. 2022) form intricate networks in the reentrant angle and more rarely along the vertical growth bands (Figure 6a,b). They consist of hollow tubes (Figure 6c) which in reflected light display a pale brownish color (Figure 6b,c), and are dotted by opaque grains of iron sulfides, few μm in diameter (Figure 6d). SEM‐EDS analysis showed a pervasive permineralization of the filament outer sheets, mostly consisting of flaky clay minerals (Figure 7a,b) enriched in Mg, K and Fe (Figure 7c,d). Some filaments are coated by μm‐sized spheroidal hollow dolomite microcrystals (Figure 7e–h), locally forming cauliflower‐like aggregates.

#### 4.1.2 Floccules

Floccules consist of organic‐ and clay‐rich aggregates with an irregular, amoeboid shape and a clotted‐peloidal structure (Figure 8a–c). These solid inclusions, up to 500 μm across, are common in all the studied lithofacies. In reflected light the floccules are pale brownish and dotted by few micron‐sized‐ opaque iron sulfides grains (Figure 8d), similar to filaments. SEM‐EDS analyses reveal that they are composed of abundant Mg‐, K‐, and Fe‐rich flaky clays (Figure 9a,b), pyrite grains (Figure 9c,d) and clusters of dolomite crystals a few microns in size (Figure 9e,f). They commonly include remains of partially dissolved and clay‐coated planktonic and benthic diatoms (Figure 9g,h).

#### 4.1.3 Spheroids

Transparent (Figure 10a,b) to reddish (Figure 10c) spheroidal objects a few μm in diameter are particularly abundant from the fourth gypsum bed upper upward. They can be isolated (Figure 10a) or clustered in chains of a few to tens of individuals (Figure 10b). They are often associated with tiny, button‐like, or squared particles of reddish color (Figure 10d). SEM‐EDS analysis highlighted a corrugated surface (Figure 10e–g) and a clayey composition typified by high values of Si and Mg, with a minor content in Ca, K, Fe, and Mn (Figure 10h).

### 4.2 CLSM Observations and Spectral Analyses

**FIGURE 11:**
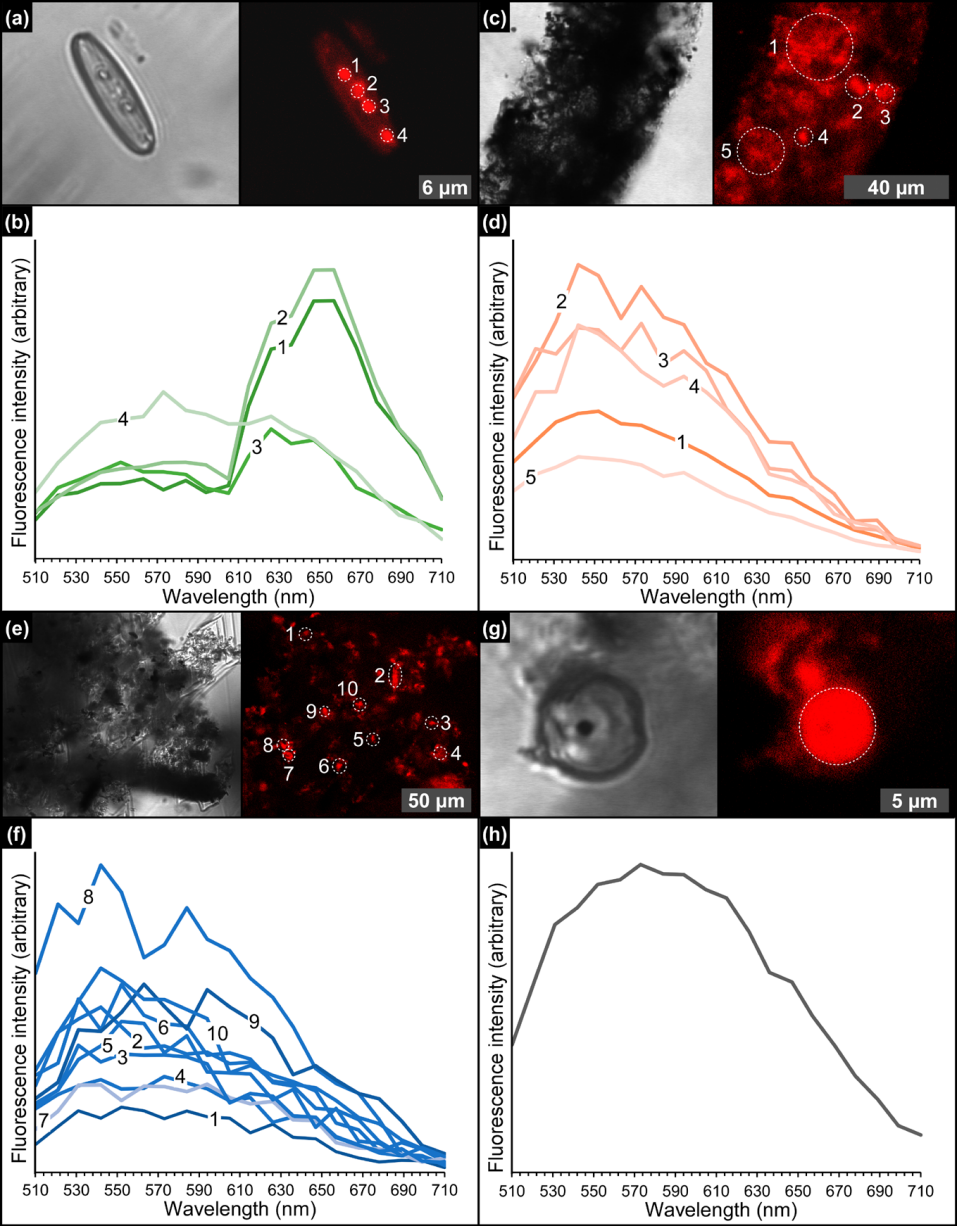
CLSM photomicrographs and spectral analyses of the solid inclusions. (a) Diatom frustule bearing autofluorescent globules and (b) corresponding emission spectrum (massive selenite—11th PLG cycle). (c) Detail of a filamentous structure and (d) and corresponding emission spectrum (massive selenite—4th PLG cycle). (e) Floccule and (f) corresponding emission spectrum (banded selenite—7th PLG cycle). (g) Spheroid and (h) corresponding emission spectrum (banded selenite—9th PLG cycle).

**FIGURE 12:**
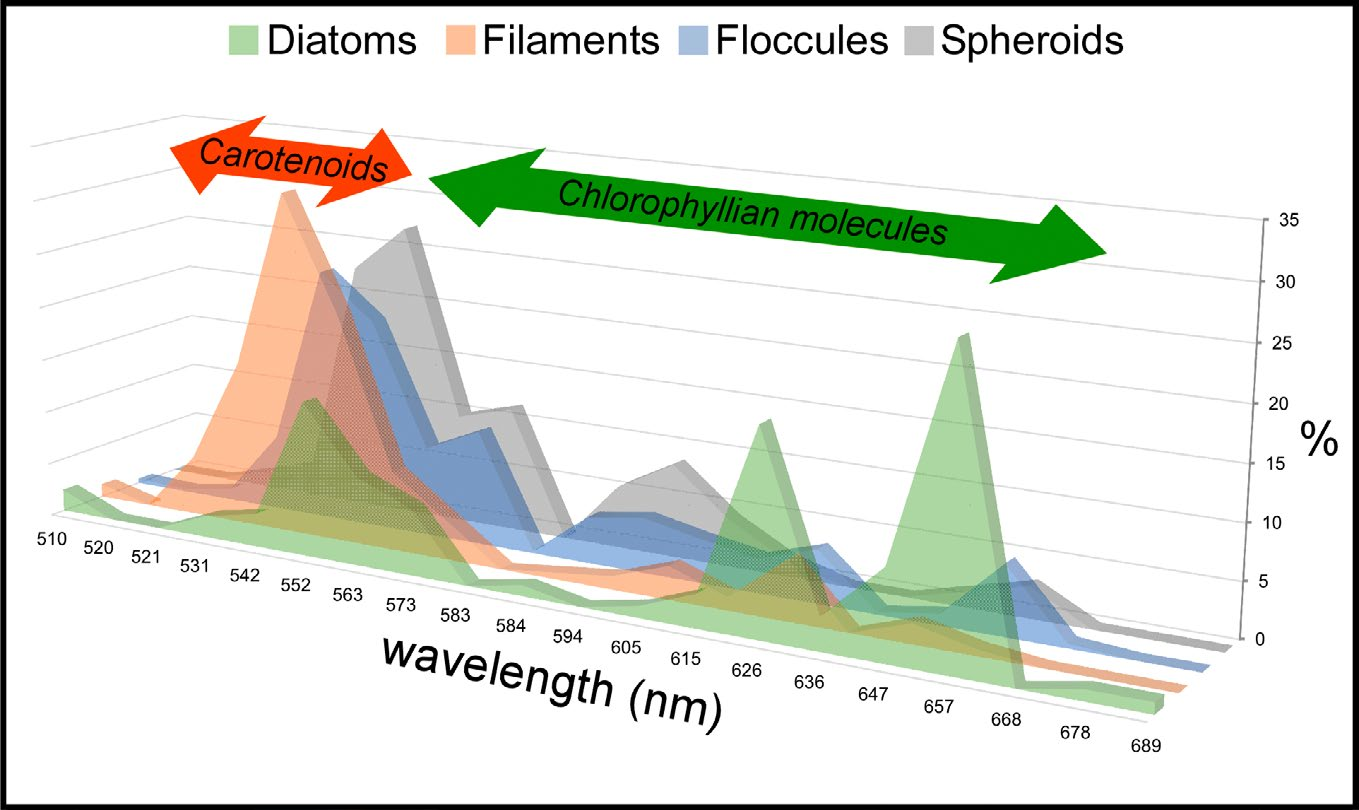
Relative percentages of regions of interest characterized by fluorescence maxima comprised between 510 and 710 nm, for each solid inclusion analyzed. The arrows highlight the wavelengths compatible with carotenoids and chlorophyllian pigments. Since no fluorescence maxima above 689 nm have been found, the 710 nm wavelength is not reported. For the sake of readability, the graph is slightly tilted with respect to the vertical axis.

Under CLSM, most of the diatom remains (Figure 11a,b), filaments (Figure 11c,d), floccules (Figure 11e,f), and spheroids (Figure 11g,h) exhibit a strong autofluorescence. Overall, diatom fluorescence maxima are mostly concentrated between 657 and 626 nm (~27% and ~18% of the regions of interest analyzed) and 552 nm (~14%) (Figure 12 and Data S1). No significant differences in fluorescence spectral values have been found between well‐preserved and altered diatom remains. In contrast, filaments, floccules, and spheroids show fluorescence maxima mostly between 542 and 552 nm (Figure 12 and Data S1).

### 4.3 Bulk Mineralogy

X‐ray powder diffraction highlighted the occurrence of five main clay mineral families, comprising in order of abundance smectite, illite, chlorite, and kaolinite, with non‐quantifiable smectite‐illite interlayers (Table 1). The clay mineral relative abundance is very similar in all the analyzed samples, with smectite always dominant (up to 69% in the banded selenite from the seventh PLG cycle) except for the first gypsum bed, which is characterized by a higher abundance of illite (60%). The samples of massive selenite from the third and the sixth cycle are both characterized by the presence of smectite‐illite interlayers.

**TABLE 1:** Bulk clay mineralogical data in relative percentage (− = absent, n.q. = not quantified due to the occurrence of smectite‐illite interlayered clays or uncertainties about the occurrence of chlorite in the sample).

PLG cycle	Lithofacies	Smectite	Illite	Smectite‐illite	Chlorite	Kaolinite
1	Massive selenite	20	60	—	16	4
3	Massive selenite	—	n.q.	n.q.	n.q.	—
6	Massive selenite	n.q.	n.q.	n.q.	n.q.	n.q.
6	Branching selenite	56	28	—	9	7
7	Massive selenite	67	11	—	5	17
7	Banded selenite	69	12	—	4	15
13	Massive selenite	59	23	—	16	2
13	Banded selenite	46	41	—	11	2
15	Massive selenite	62	18	—	2	18
16	Massive selenite	62	17	—	4	17

## 5 Discussion

### 5.1 Excellent Biosignature Preservation in Messinian Gypsum

**FIGURE 13:**
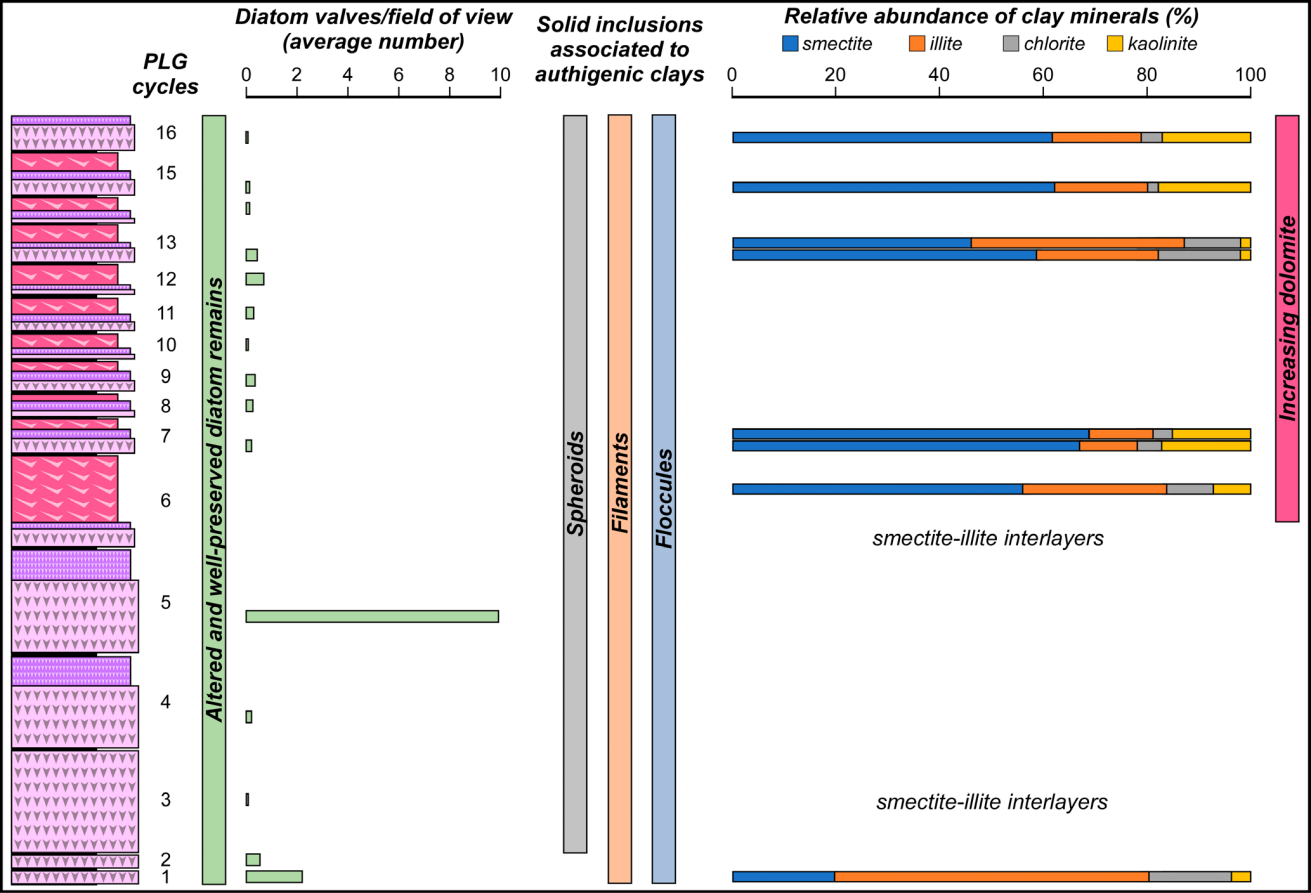
Summary of the main results.

The gypsum crystals of the massive selenite lithofacies from all the beds are typified by abundant diatom remains (Figure 13). In contrast, in the banded and branching selenite lithofacies, diatoms are less abundant or even absent. The nano‐sized planktonic taxa (Figure 3a,b,e), especially abundant in the lower gypsum beds, suggest deposition in a stratified (and relatively) deep basin with brackish to normal marine salinity conditions in the upper water column (e.g., Pellegrino et al. 2021). Benthic diatoms mostly represented by naviculoids (Figure 3c,d,f) become dominant from the third gypsum bed upwards, possibly suggesting the shallowing of the basin from out of the photic zone (lower two gypsum beds) to the photic zone. Indeed, naviculoid diatoms are often observed in shallow environments characterized by wide salinity fluctuations (e.g., Bae et al. 2020). The state of preservation of diatom remains is variable. Some of the best‐preserved remains display tiny reddish to yellowish globules of carbonaceous material (D and G bands in Raman spectra; Figure 4) with a strong autofluorescence in a range compatible with chlorophyll and its derivatives like chlorins and pheophytins (~680–550 nm) or carotenoids (~550–500 nm) (Figures 11a,b and 12; e.g., Lepot et al. 2014; Giampaoli et al. 2020; Wolkenstein and Arp 2021; Bunkin et al. 2023). These features possibly correspond to lipid droplets, which in living diatom cells are closely associated with chloroplasts (e.g., Leyland, Boussiba, and Khozin‐Goldberg 2020; Guéguen et al. 2021). Alternatively, they might represent shrunken chloroplasts, which are commonly observed in diatoms under osmotic stress or nitrogen deprivation (e.g., Maeda et al. 2017). Irrespective of their origin, the preservation of putative diatom cell ultrastructures with associated pigments, only rarely reported in the geologic record (e.g., Stasiuk and Sanei 2001; Wolfe et al. 2006), suggests that the entrapment of diatom remains in gypsum has occurred very rapidly, before the complete degradation of cellular ultrastructures and pigment molecules. Experimental studies indicate that in the case of planktonic diatoms, cell death and subsequent lysis occur in less than a week after they have sunk out of the photic zone (e.g., Agustí et al. 2018). Such observation confirms the very high potential of gypsum in preserving signatures of past life (e.g., Schopf et al. 2012; Benison and Karmanocky III 2014). In this sense, not only halite but also gypsum should be considered as a potential Lagerstätte (e.g., Gibson and Benison 2023).

### 5.2 Early Diagenesis and Authigenic Mineral Formation

#### 5.2.1 Biogenic Silica Alteration

**FIGURE 14:**
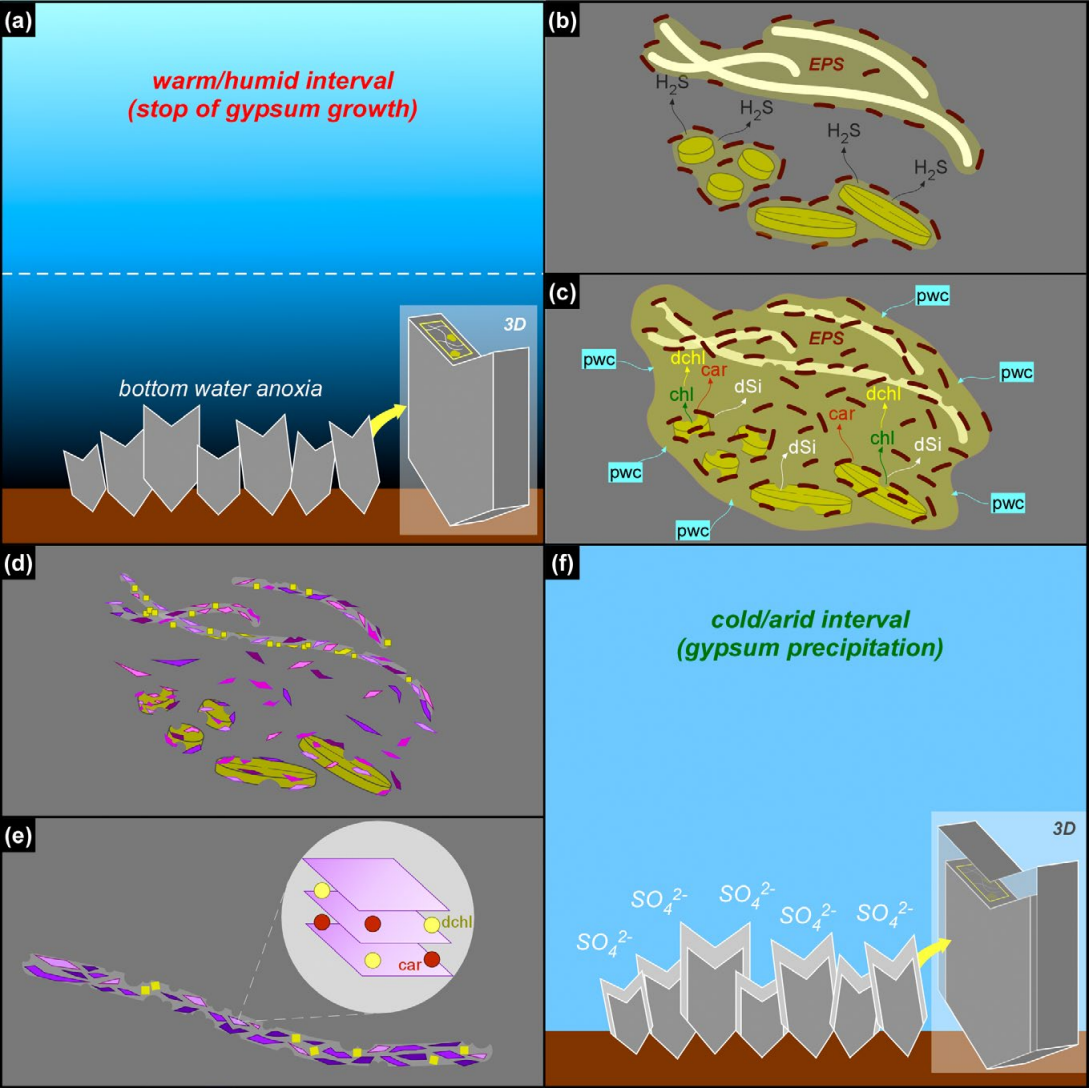
Simplified sketch of the diagenetic processes discussed in the text. For sake of simplicity, only the massive selenite lithofacies and two types of solid inclusions (diatoms and filaments) are illustrated. (a) During warm/humid phases, increasing freshwater input favors nutrient input and water column stratification, with the consequent establishment of bottom water anoxia. (b) Sulfate‐reducing bacteria (dark brown) degrades EPS‐rich aggregates of filaments and diatoms, and favors the build‐up of hydrogen sulfide in bottom waters. (c) Sulfidic conditions enhanced diatom cell lysis and the release of organic matter in pore waters, comprising pigments (chl = chlorophyll, car = carotenoids); chlorophyll is rapidly degraded (dchl = degraded chlorophyll), while carotenoids are more refractory to degradation; diatom cell lysis favors frustule dissolution, with consequent build‐up of dissolved silica (dSi) in the pore waters; dSi combines with pore water cations (pwc), attracted by the negatively‐charged EPS matrix produced by bacterial proliferation. (d) Neoformed clays (purple polygons), which together with dolomite (yellow squares) pervasively coats filaments. (e) The neoformed clays entrap carotenoids and degraded chlorophyllian pigments in their interlayers. (f) Formation of a new gypsum lamina.

Other diatom remains display strong evidence of alteration, consisting of opal dissolution and authigenic clay overgrowth (Figures 5 and 9g,h) that took place before the entrapment of the biogenic material in gypsum. In particular, considering the fine lamination observed in the crystals, which reflects cyclical change in the gypsum saturation state under the control of seasonal climate oscillations (e.g., Reghizzi et al. 2018), these processes must have occurred when gypsum growth was interrupted temporarily by enhanced continental runoff (Figure 14a). Runoff resulted in water column eutrophication fueling the bloom of planktonic and benthic diatoms; subsequent density stratification of water column in combination with decaying organic matter promoted bottom anoxia, favoring organoclastic sulfate reduction (OSR) (e.g., Dela Pierre et al. 2015). OSR, in turn, may have strongly influenced biogenic silica alteration in different ways, possibly interlinked (Figure 14b,c). First, OSR may have exposed biogenic silica to degradation by removing the organic coat enveloping the frustules, a process requiring diatom cell death (e.g., Moriceau et al. 2014). The establishment of sulfidic conditions promoted by active OSR may have enhanced diatom cell lysis and the consequent release of the organic content of frustules (e.g., Ciglenečki et al. 2003). In the gypsum samples studied herein the initial degradation of diatom cells induced by OSR may have resulted in a runaway effect: the increasing production of H_2_S, poisoning the seafloor where planktonic diatoms sank and benthic diatoms thrived, likely triggered a widespread cell lysis that favored the release of organic matter, further fueling the OSR.

Second, OSR may have enhanced dissolution of biogenic silica by raising pore water pH (e.g., Wright 1997; Pellegrino et al. 2023). The availability of dissolved silica derived from opal dissolution and pore water cations, promoted clay neoformation on altered diatom remains and elsewhere in the sedimentary environment (e.g., Pellegrino et al. 2023; Figure 14b–d).

Interestingly, diatom remains with preserved putative cellular ultrastructures (Figures 3c,d and 11a) coexist in the same samples with altered diatom remains (Figure 5), raising the question on how the former escaped the degradative processes that affected the latter. Different explanations can be proposed: (i) unaltered planktonic and benthic diatoms were deposited or thrived in microenvironments typified by transient oxygenation; these diatoms were not affected by H_2_S poisoning, and therefore escaped cell lysis before their final inclusion in gypsum, which may have occurred when they were still alive; (ii) the diatoms could have survived bottom water anoxia thanks to nitrate reduction (e.g., Kamp et al. 2010), or (iii) the rate of diatom productivity was so sustained to outcompete the rate of degradative processes, allowing the excellent preservation of a smaller group of survivals immediately entrapped by gypsum growth.

#### 5.2.2 Filaments, Floccules, and Spheroids: Further Substrates for Clay Neoformation

Filamentous microfossils are coated by flaky clays enriched in Mg, K, and occasionally Fe (Figure 7a–d), which are clearly nucleated onto the organic template provided by the precursor organism (see also Dela Pierre et al. 2015). The original biomass was removed, leaving hollow tubes (Figure 6c) that were subsequently coated with authigenic minerals derived from microbial‐mediated processes at the expense of biogenic silica. Clay mineral neoformation was likely prompted by the availability of dissolved silica provided by the degradation of biosiliceous remains and of pore water cations (e.g., Suosaari et al. 2022; Pellegrino et al. 2023), as already discussed for diatoms (Figure 14c,d). Similar processes may have occurred in floccules and in spheroids. Floccules correspond to organo‐mineral aggregates formed in the water column (marine snow floccules; e.g., Dela Pierre et al. 2014) or at the seafloor, through the binding of extracellular polymeric substances (EPS) associated with biogenic particles with clay‐ and silt‐sized terrigenous grains (e.g., Vos, de Boer, and Misdorp 1988). In this case, the distinction between terrigenous and neoformed clays is not straightforward, but the feathery appearance and honeycomb structure of a great part of the clays included in floccules (Figure 9a), as well as the presence of altered diatom remains (Figure 9g,h), is in favor of the authigenic origin of most of the clayey material. Abundant EPS sourced by bacteria and diatoms likely acted as a template for clay precipitation (e.g., Bashkar et al. 2005; Benson, Smith, and Spaulding 2021; Novis et al. 2021; Suosaari et al. 2022; Mather et al. 2023).

The origin of spheroids (Figure 10), reported for the first time from Messinian gypsum, is still not clear. The size and coccoid morphology are shared by a wide array of planktonic and benthic prokaryotes, especially some cyanobacteria (e.g., Komárek and Johansen 2015). Nevertheless, similar objects have been experimentally obtained from solutions enriched in sulfide, further oxidized in the presence of organic molecules and can therefore be of abiotic origin (e.g., Nims et al. 2021).

To summarize, the fact that non‐diatomaceous biogenic remains preserved in gypsum are coated by authigenic clays mostly of smectitic composition, implies the large availability of dissolved silica in pore waters and therefore the early diagenetic dissolution of diatomaceous material (e.g., Badaut and Risacher 1983; del Buey, Sanz‐Montero, and Sánchez‐Román 2023) occurring before its entombment in gypsum.

### 5.3 Did Neoformed Clays Bind Diatom‐Derived Pigments?

The autofluorescent signal of diatom remains (Figures 11 and 12) covers a wide range of wavelength maxima, compatible with the very early (650–600 nm) to early (600–550 nm) byproducts of chlorophyll degradation, like the pheophytins, as well as carotenoids (550–500 nm) (e.g., Giampaoli et al. 2020). In contrast, non‐diatomaceous solid inclusions coated by neoformed clays (filaments, floccules, and spheroids) display peaks compatible with carotenoid fluorescence spectra. Pheophytins derive from chlorophyll demetallation (i.e., the first stage of the degradative pathway of the chlorophyll molecule), consisting in the removal of Mg^2+^ from the tetrapyrrole ring (e.g., Louda et al. 1998; Giampaoli et al. 2020). For what concerns carotenoids, these compounds are more refractory to degradation than chlorophyll, due to their simpler molecular structure (e.g., Leavitt and Hodgson 2002). However, little is still known about the impact of early diagenetic processes on pigment degradation (cfr. Belle et al. 2022).

The formation of the authigenic clays requires the early diagenetic dissolution of biogenic silica, as discussed above. Besides the build‐up of dissolved silica in pore waters, diatom cell lysis may have caused the copious release of organic matter in the sedimentary environment, including pigments (Figure 14c). We suggest that such diatom‐derived organic matter was adsorbed in the interlayers of authigenic clays (Figure 14e), which acted as stabilizers (e.g., Kennedy, Pevear, and Hill 2002; Kennedy et al. 2014; Li, Mu, et al. 2021; Zhao, Xu, and Hao 2023). In this light, it is possible that the autofluorescent signal observed in the clay‐coated, non‐diatomaceous solid inclusions (filaments, floccules, and spheroids) derives from organic matter released by diatoms (or other phototrophs that did not leave a fossil record) and does not necessarily reflect the original biomass of the precursor organisms.

Summarizing, the above evidence suggests that the degradation of diatom remains during the temporary interruption of gypsum growth may have contributed to the release of diatom cell organic matter, comprising photosynthetic pigments. We suggest that such pigments, after being partially degraded, were adsorbed onto authigenic clays formed on non‐diatomaceous solid inclusions (filaments, floccules, and spheroids), which therefore preserved a new biosignature. The periodical growth of gypsum interrupted these processes (Figure 14f), entrapping both excellently preserved and severely altered biosignatures.

### 5.4 Implications for the Messinian Salinity Crisis

The results of this study highlight that toward the top of the VdG section, the reduction or disappearance of diatom remains coincides with an increase of Mg‐bearing minerals, such as dolomite and smectite (Figure 13). High contents of dolomite and smectite in the MSC sediments have been originally interpreted as reflecting sabkha‐like conditions (e.g., Friedman 1973) and the periodical denudation of peri‐Mediterranean soils developed under a semi‐arid climate (e.g., Chamley, Giroud d'Argoud, and Robert 1977), respectively.

Actually, the branching selenite lithofacies, which appears from the sixth PLG cycle upwards (Figures 1c and 13), is typified by abundant dolomite microcrystals, not observed in the massive and banded selenite. The habit of dolomite microcrystals supports their microbial origin, possibly induced by OSR in anoxic pore waters (e.g., Natalicchio et al. 2021). Interestingly, the appearance of the branching selenite lithofacies at 5.84 Ma coincides with a marked deviation of Sr isotopes from global ocean values (e.g., Reghizzi et al. 2018; Figure 1c), coupled with the progressive shallowing of the basin suggested by the stacking pattern of the gypsum beds (e.g., Lugli et al. 2010; Natalicchio et al. 2021). Such circumstance has suggested that the VdG basin experienced a more pronounced restriction starting from 5.84 Ma onward, becoming more sensitive to continental runoff (Reghizzi et al. 2018). Such a scenario may have further enhanced the periodical stratification of water column and bottom water anoxia, increasing the impact of the diagenetic processes at the expense of organic matter and biogenic silica, as described above (e.g., Pellegrino et al. 2023).

Basin confinement and enhanced OSR may have increased the availability of critical elements for the in situ formation of Mg‐bearing minerals. Experimental studies suggested that both sulfidic and silica‐rich environments may catalyze dolomite precipitation by lowering the energy barrier required for dehydrating Mg^2+^ ions, making them available for the incorporation into the carbonate crystal lattice (e.g., Zhang et al. 2012; Fang et al. 2023). Other authors highlighted the fundamental role played by organic compounds, like EPS, in promoting dolomite precipitation (e.g., Petrash et al. 2017; Diloreto et al. 2021; Li, Wignall, et al. 2021). Similar mechanisms have been suggested for explaining the widespread precipitation of smectitic clays in diatom‐dominated microbial mats (e.g., del Buey, Sanz‐Montero, and Sánchez‐Román 2023). We suggest that the degradation of Mg‐bearing biomolecules, notably chlorophyll, provided an additional source of Mg^2+^ ions for both dolomite and smectitic clay formation (e.g., Gebelein and Hoffman 1973; Wright 1997). In this light, the formation of Mg‐rich minerals associated with Messinian gypsum might have been enhanced by the availability of organic matter and dissolved silica sourced by diatom degradation.

## 6 Conclusions

The results of this study highlight the co‐occurrence, within the same gypsum crystal, of superbly preserved and severely altered biogenic remains. Such puzzling scenario demonstrates that the extreme environment into which Messinian gypsum formed was characterized by high frequency (sub‐annual?) fluctuations of bottom water chemistry. Such variability drastically modified the preservation potential of biogenic remains, which shifted from preservation‐prone, when arid conditions favored gypsum precipitation, to preservation‐hostile, when humidity, water column stratification and bottom water anoxia interrupted gypsum growth and induced microbially mediated, very early diagenetic processes at the expense of organic matter and associated biominerals, in particular diatom biogenic silica. The consequent build‐up of dissolved silica, in combination with pore water cations, contributed to the formation of authigenic clays. The latter acted as traps for the photosynthetic pigments released by diatom cell lysis. Such process was furtherly enhanced by more severe basin restriction and bottom anoxia initiated at ~5.84 Ma, when the branching selenite lithofacies appeared. This confirms the hypothesis of a significant diagenetic bias affecting the Messinian biogenic sedimentary record, associated with the establishment of the Mediterranean Salt Giant. At the same time, these findings highlight that the linkage between biomolecules and precursor organism is not always straightforward, and need to be evaluated through detailed petrographic analyses aimed at the identification of authigenic minerals potentially revealing the intervention of diagenetic processes redistributing organic matter in the sedimentary environment, with significant implications for near future astrobiological investigations of Martian sediments (e.g., McMahon et al. 2018; Benison et al. 2024; Siljeström et al. 2024).

## Conflicts of Interest

The authors declare no conflicts of interest.

## Supporting information


Data S1.

## Data Availability

The data that support the findings of this study are available from the corresponding author upon reasonable request.
