# Supplementary material for: The Impact of Early Diagenesis on Biosignature Preservation in Sulfate Evaporites: Insights From Messinian (Late Miocene) Gypsum
Source: Geobiology. 2024 Dec 9;22(6):e70007. doi: 10.1111/gbi.70007 (PMC11629073; doi:10.1111/gbi.70007)
Supplement: Supplementary file 1 — Data S1. [file GBI-22-e70007-s001.docx]

Supporting Information

**PROTOCOL ADOPTED FOR BULK MINERALOGICAL ANALYSIS**

About 10 g of each sample was ground with a pestle in an agate mortar until a particle size less than 500 µm was achieved. The resulting powder was placed into a beaker filled with 600 mL of ultrapure water. A stir bar was added, the beaker placed onto a magnetic stirrer, the solution stirred at 4000 rpm for 5 minutes and left to settle for 3 hours. A pipette was used to remove the topside 5 cm of the solution still retaining the fine, clay-rich fraction. Then, the recovered solution was filtered onto a nitrocellulose filter (0.45 µm pore size). This procedure was repeated 8 times for each powdered sample, in order to obtain enough clayey fraction for performing the mineralogical analysis. After scraping the filters, the recovered fraction was placed into a centrifuge tube filled with 45 mL of ultrapure water, then dispersed for 2 minutes with the ultrasonic probe. The <2µm grain-size fraction was separated by centrifugation and the centrifugation time calculated by applying the Stokes’ law to a simplified spherical particle model. After centrifugation the suspension containing the <2µm grain-size fraction was transferred into a vacuum filter apparatus, then filtered onto a nitrocellulose filter. Oriented slides of clay-rich film for XRD analyses were prepared by the filter transfer method described by Moore & Reynolds (1997).

X-ray diffraction analyses was performed with a Rigaku-Miniflex 600 XRD system (CuKα radiation) operating at 40 kV and 15 mA. Oriented, air dried, clay-rich films were scanned from 2° to 65° 2θ (step size of 0.04° and speed of 0.5°/min). The same samples were scanned after treatment with ethylene glycol (EG, hereinafter) and then heated at 550°C. EG solvated and heated samples were scanned at the same operating conditions as air-dried aggregates, with a scanning interval of 2°-20° 2θ.

The reflection expanding from 14.6 Å to 16.9 Å upon glicolation belongs to the smectite (S) mineral family. We assigned this peak to the (001) reflection of the montmorillonite, as a reference of smectite family for the semi-quantitative analysis. Clay mineral peaks expanding to values less than 16.9 Å upon glycolation are assigned to an Illite/EG-smectite mixed layer. According to Moore & Reynolds (1997), the percentage of illite in illite/EG-smectite mixed layer has been calculated by using the °Δ2θ method after decomposing the peaks between 9°–10° and 16°–17° 2θ using Fityk software program (Wojdyr, 2010) with Voight function. Illite (I) is identified by the (001) reflection located at 10 Å and not affected by EG solvation or heat treatment. Chlorite (C) is identified by the weak (001) reflection raising at 14.0 Å (6.3°2θ) after heat treatment to 550°C, and by the (004) reflection at 3.54 Å. Kaolinite (K) is univocally identified by the (002) reflection at 3.58 Å (24.9 °2θ).

When mixed layers were not detected in the sample, a semiquantitative analysis was performed on air dried XRD patterns by exploiting the calculated mineral reference intensities and the related mineral intensity factor (MIF) for the (003) of montmorillonite, (002) of illite, (003) of chlorite, and (002) of kaolinite, according to the procedure described in Moore & Reynolds (1997). The (003) reflection of illite was used as a reference intensity to calculate the MIF factor. In addition, the calculated mineral reference intensities of the clays reflection just mentioned above were multiplied by an appropriate value of sinθ because a θ-compensating slit, exploited in our measurements, causes an intensity variation that is proportional to sinθ (Moore & Reynolds, 1997). Fityk software with Voight function was used to decompose the peaks investigated for semi-quantitative analysis, calculating the related area value (Wojdyr, 2010).

**References**

Moore, D.M., Reynolds, R.C. Jr., 1997. X-ray diffraction and the identification and analysis of clay minerals. Oxford University Press (2^nd^ edition), 378 pp.

Wojdyr, M., 2010. Fityk: a general-purpose peak fitting program. J. Appl. Cryst. 43 (5), 1126-1128. <https://doi.org/10.1107/S0021889810030499>

**XRD SPECTRA**

**1^st^ PLG cycle – Massive selenite**

**
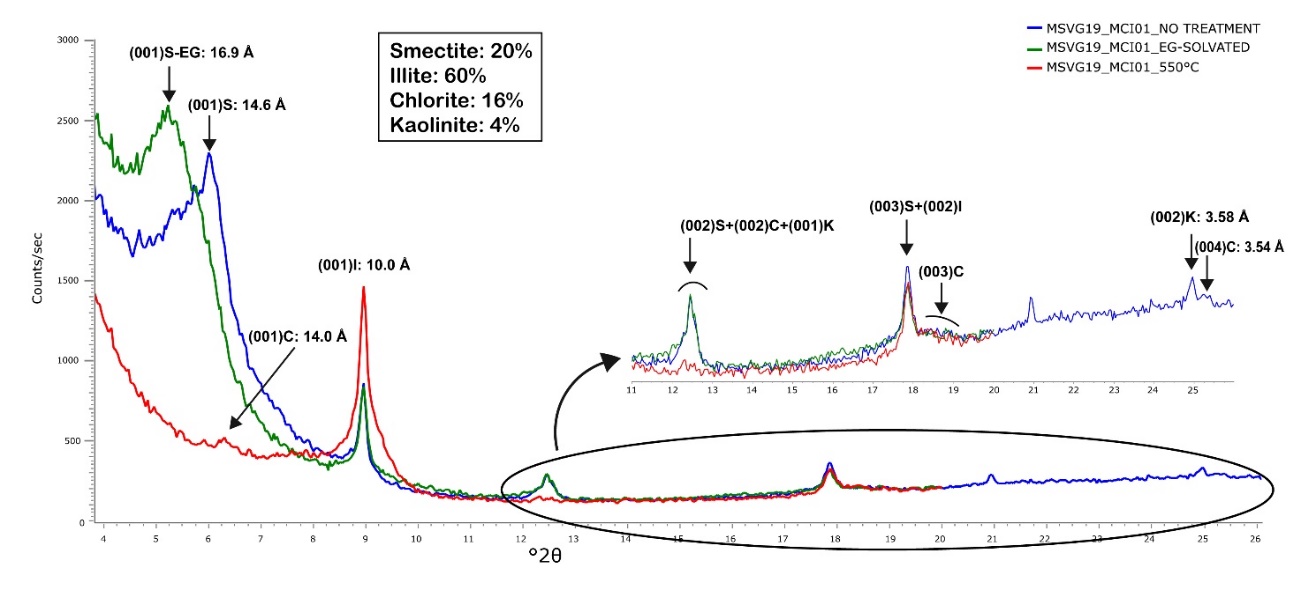
**

**3^rd^ PLG cycle – Massive selenite**

**
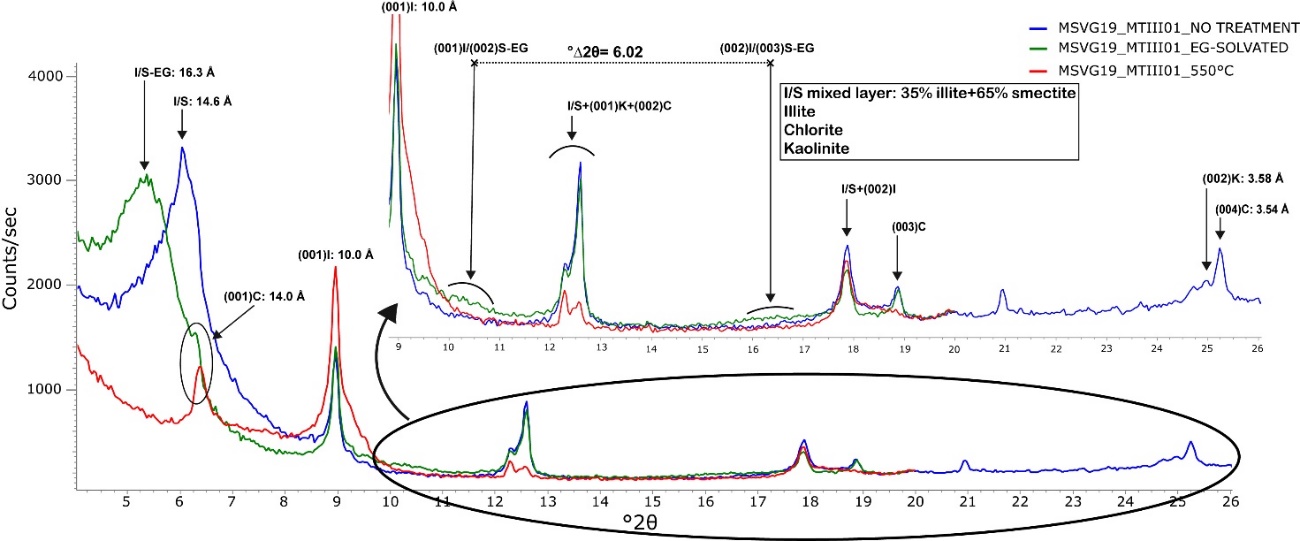
**

**6^th^ PLG cycle – Massive selenite**

**
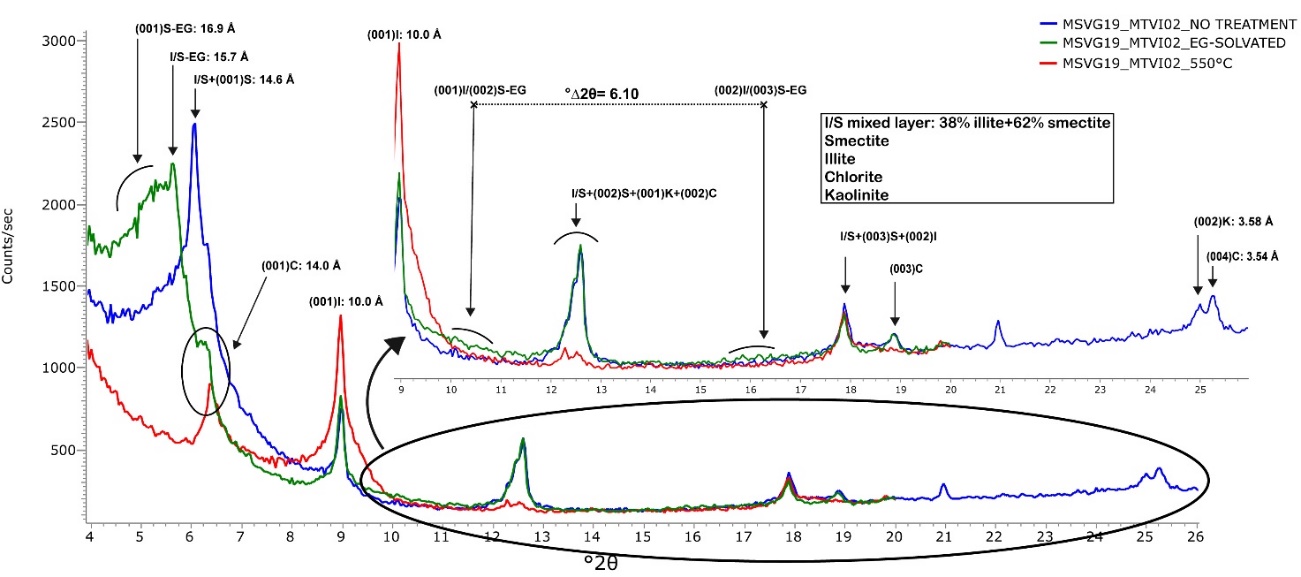
**

**6^th^ PLG cycle – Branching selenite**


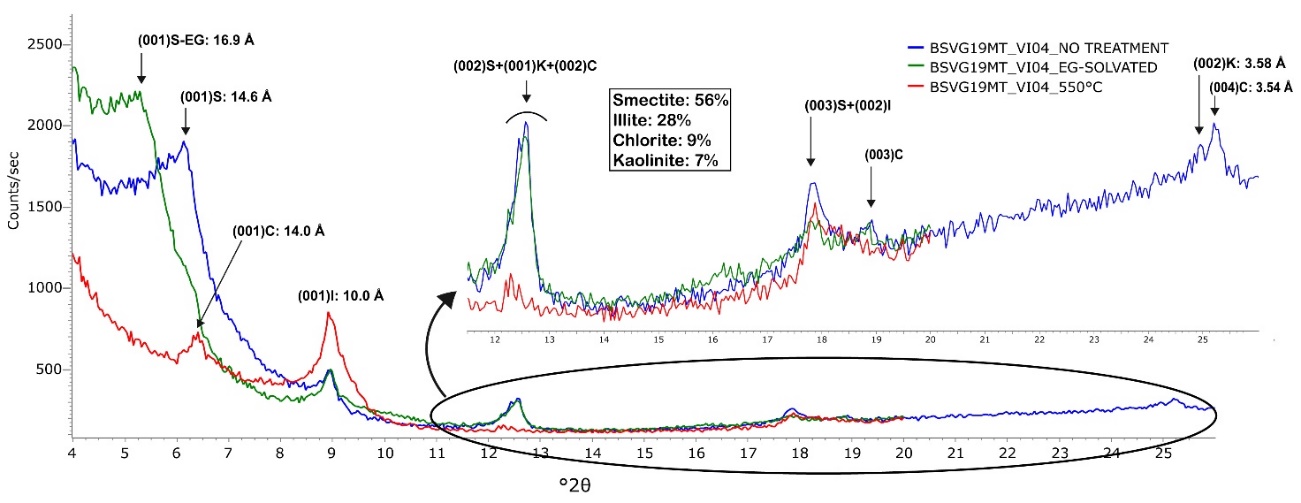


**7^th^ PLG cycle – Massive selenite**

**
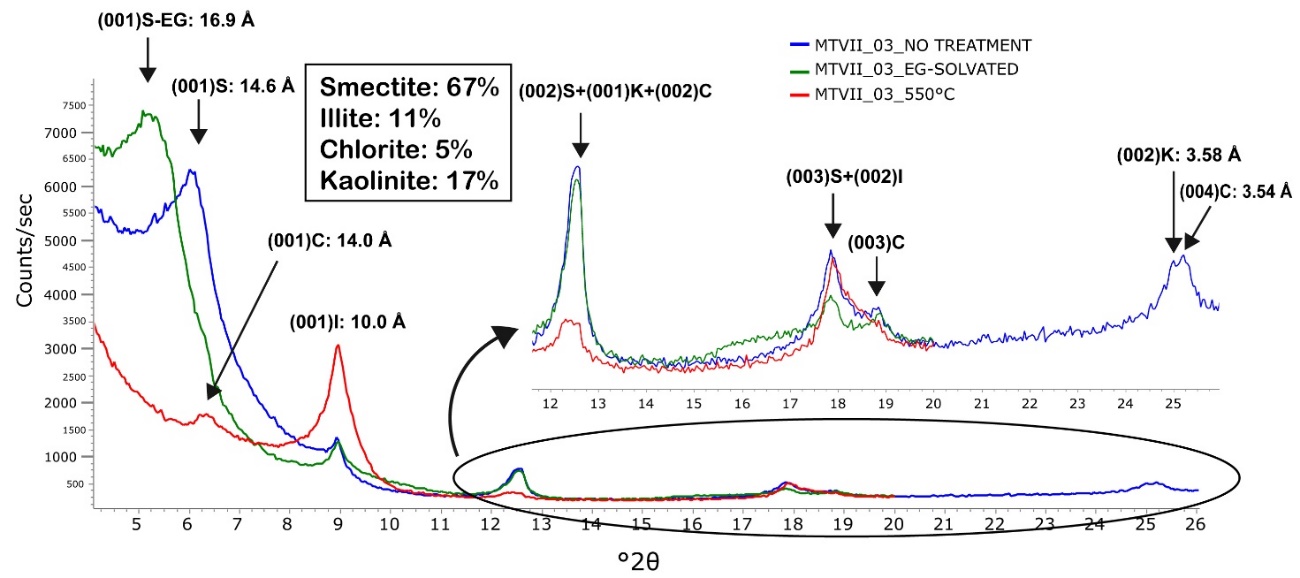
**

**7^th^ PLG cycle – Banded selenite**

**
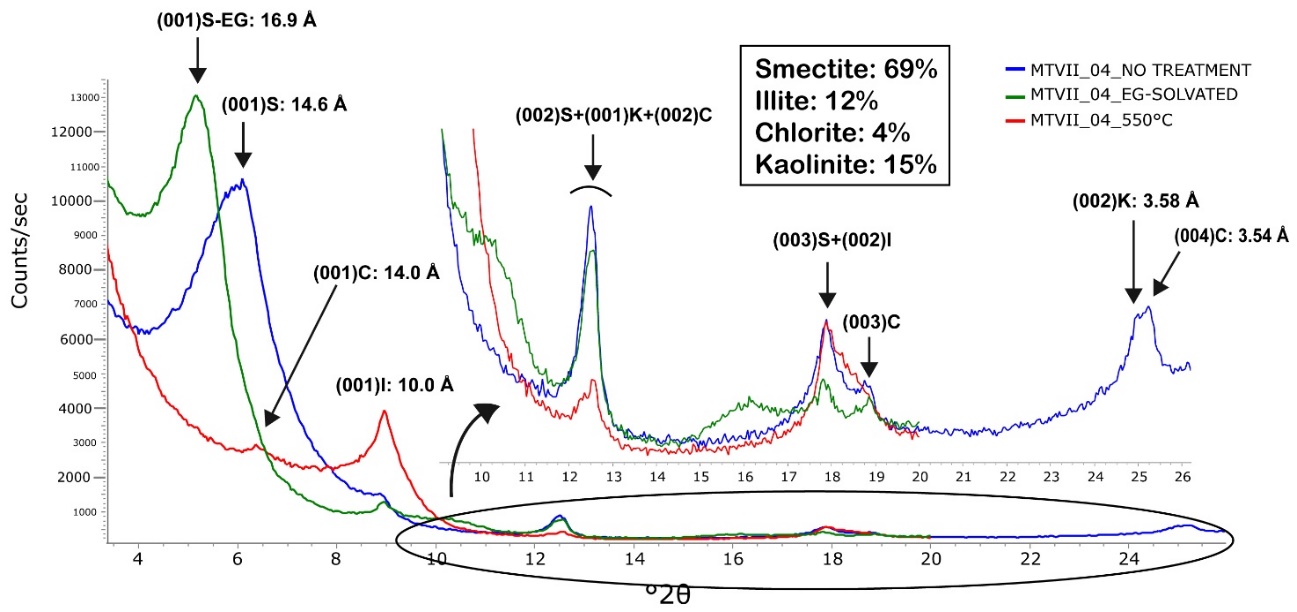
**

**13^th^ PLG cycle – Massive selenite**


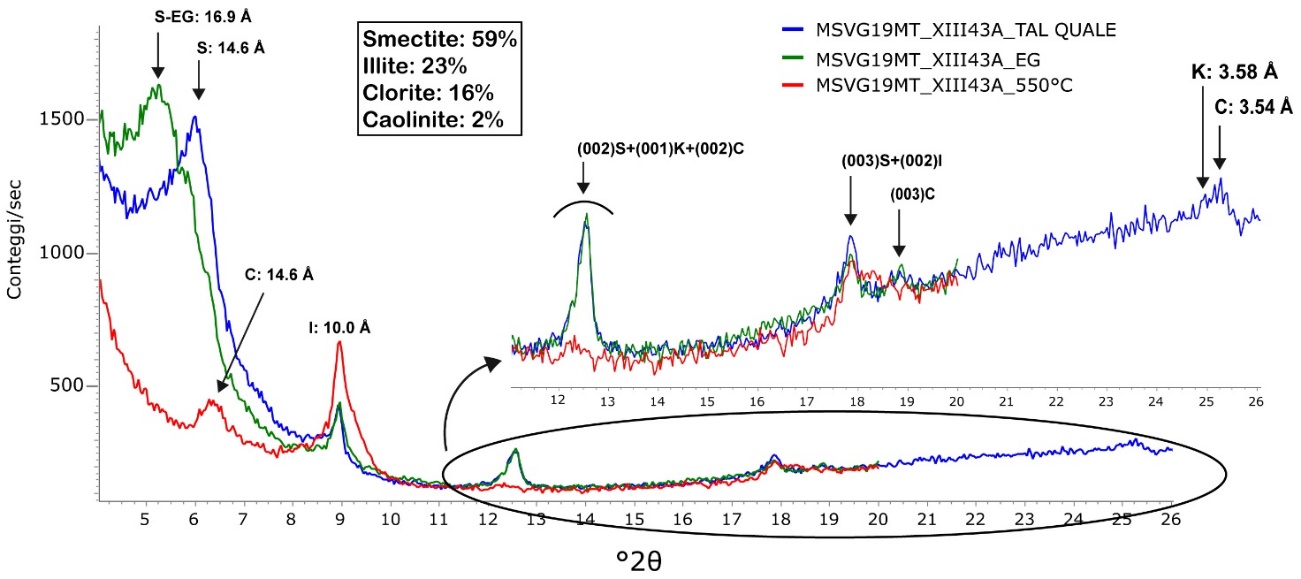


**13^th^ PLG cycle – Banded selenite**


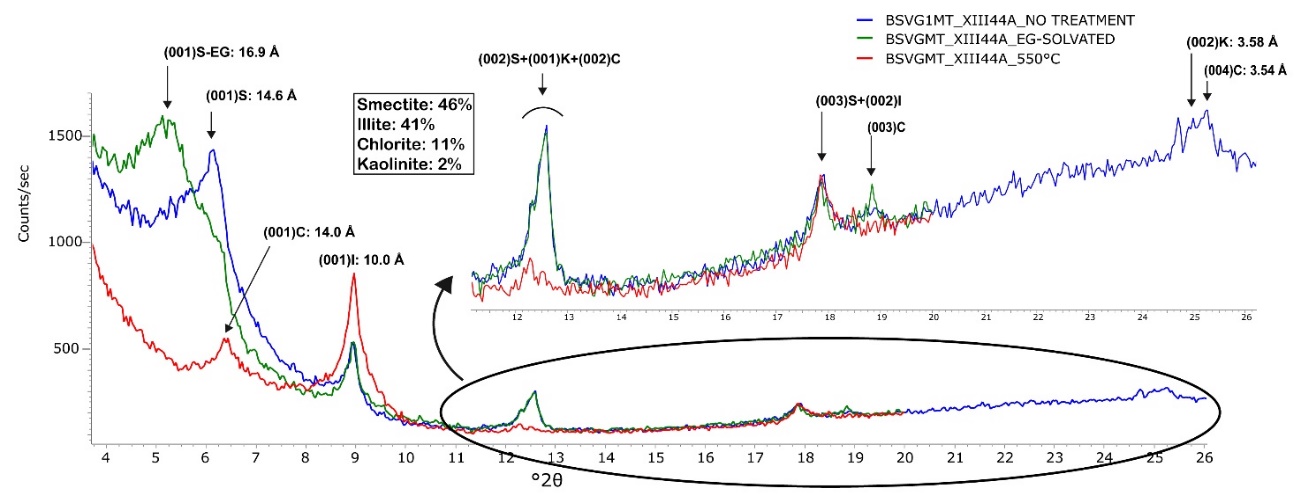


**15^th^ PLG cycle – Massive selenite**

**
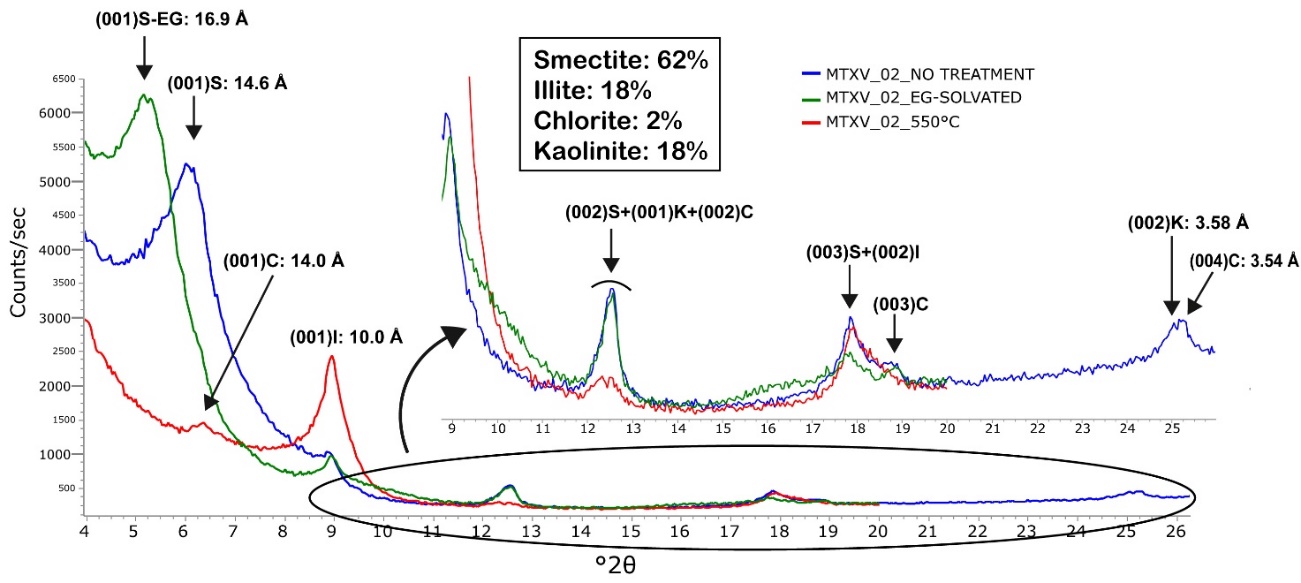
**

**16^th^ PLG cycle – Massive selenite**

**
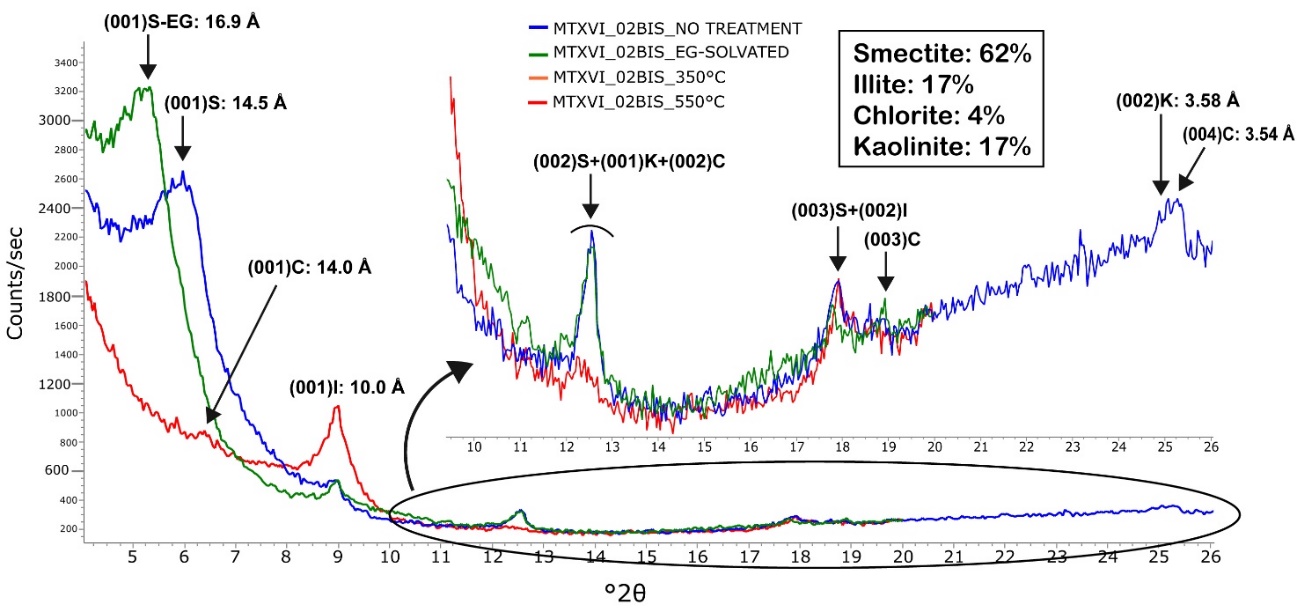
**

**CLSM SPECTRAL ANALYSIS**

The following table resumes the spectral analysis dataset. For each type of solid inclusion, the number (n) and relative percentage (%) of the regions of interest (ROI) exhibiting fluorescence maxima at specific wavelengths (λ) are reported.

| **λ (nm)** | **Diatom remains**  **(Analyzed ROI = 98)** | | **Filaments  (Analyzed ROI = 164)** | | **Floccules**  **(Analyzed ROI = 473)** | | **Spheroids**  **(Analyzed ROI = 70)** | |
| --- | --- | --- | --- | --- | --- | --- | --- | --- |
|  | **n** | **%** | **n** | **%** | **n** | **%** | **n** | **%** |
| **689** | 1 | 1.02 | 0 | 0.00 | 0 | 0.00 | 0 | 0.00 |
| **678** | 1 | 1.02 | 0 | 0.00 | 0 | 0.00 | 0 | 0.00 |
| **668** | 0 | 0.00 | 0 | 0.00 | 2 | 0.42 | 0 | 0.00 |
| **657** | 27 | 27.55 | 1 | 0.61 | 30 | 6.34 | 2 | 2.86 |
| **647** | 8 | 8.16 | 3 | 1.83 | 5 | 1.06 | 1 | 1.43 |
| **636** | 3 | 3.06 | 0 | 0.00 | 1 | 0.21 | 0 | 0.00 |
| **626** | 18 | 18.37 | 9 | 5.49 | 23 | 4.86 | 0 | 0.00 |
| **615** | 3 | 3.06 | 2 | 1.22 | 15 | 3.17 | 1 | 1.43 |
| **605** | 1 | 1.02 | 5 | 3.05 | 19 | 4.02 | 3 | 4.29 |
| **594** | 0 | 0.00 | 2 | 1.22 | 24 | 5.07 | 6 | 8.57 |
| **584** | 1 | 1.02 | 1 | 0.61 | 20 | 4.23 | 4 | 5.71 |
| **583** | 0 | 0.00 | 0 | 0.00 | 1 | 0.21 | 0 | 0.00 |
| **573** | 6 | 6.12 | 6 | 3.66 | 50 | 10.57 | 8 | 11.43 |
| **563** | 8 | 8.16 | 12 | 7.32 | 39 | 8.25 | 7 | 10.00 |
| **552** | 14 | 14.29 | 35 | 21.34 | 92 | 19.45 | 19 | 27.14 |
| **542** | 3 | 3.06 | 52 | 31.71 | 111 | 23.47 | 16 | 22.86 |
| **531** | 2 | 2.04 | 25 | 15.24 | 33 | 6.98 | 2 | 2.86 |
| **521** | 0 | 0.00 | 9 | 5.49 | 5 | 1.06 | 1 | 1.43 |
| **520** | 0 | 0.00 | 0 | 0.00 | 1 | 0.21 | 0 | 0.00 |
| **510** | 2 | 2.04 | 2 | 1.22 | 2 | 0.42 | 0 | 0.00 |
